# Evidence for an $\eta _c(1S) \pi ^-$ resonance in $B^0 \rightarrow \eta _c(1S) K^+\pi ^-$ decays

**DOI:** 10.1140/epjc/s10052-018-6447-z

**Published:** 2018-12-17

**Authors:** R. Aaij, C. Abellán Beteta, B. Adeva, M. Adinolfi, C. A. Aidala, Z. Ajaltouni, S. Akar, P. Albicocco, J. Albrecht, F. Alessio, M. Alexander, A. Alfonso Albero, G. Alkhazov, P. Alvarez Cartelle, A. A. Alves, S. Amato, S. Amerio, Y. Amhis, L. An, L. Anderlini, G. Andreassi, M. Andreotti, J. E. Andrews, R. B. Appleby, F. Archilli, P. d’Argent, J. Arnau Romeu, A. Artamonov, M. Artuso, K. Arzymatov, E. Aslanides, M. Atzeni, B. Audurier, S. Bachmann, J. J. Back, S. Baker, V. Balagura, W. Baldini, A. Baranov, R. J. Barlow, S. Barsuk, W. Barter, F. Baryshnikov, V. Batozskaya, B. Batsukh, A. Battig, V. Battista, A. Bay, J. Beddow, F. Bedeschi, I. Bediaga, A. Beiter, L. J. Bel, S. Belin, N. Beliy, V. Bellee, N. Belloli, K. Belous, I. Belyaev, E. Ben-Haim, G. Bencivenni, S. Benson, S. Beranek, A. Berezhnoy, R. Bernet, D. Berninghoff, E. Bertholet, A. Bertolin, C. Betancourt, F. Betti, M. O. Bettler, M. van Beuzekom, Ia. Bezshyiko, S. Bhasin, J. Bhom, S. Bifani, P. Billoir, A. Birnkraut, A. Bizzeti, M. Bjørn, M. P. Blago, T. Blake, F. Blanc, S. Blusk, D. Bobulska, V. Bocci, O. Boente Garcia, T. Boettcher, A. Bondar, N. Bondar, S. Borghi, M. Borisyak, M. Borsato, F. Bossu, M. Boubdir, T. J. V. Bowcock, C. Bozzi, S. Braun, M. Brodski, J. Brodzicka, A. Brossa Gonzalo, D. Brundu, E. Buchanan, A. Buonaura, C. Burr, A. Bursche, J. Buytaert, W. Byczynski, S. Cadeddu, H. Cai, R. Calabrese, R. Calladine, M. Calvi, M. Calvo Gomez, A. Camboni, P. Campana, D. H. Campora Perez, L. Capriotti, A. Carbone, G. Carboni, R. Cardinale, A. Cardini, P. Carniti, L. Carson, K. Carvalho Akiba, G. Casse, L. Cassina, M. Cattaneo, G. Cavallero, R. Cenci, D. Chamont, M. G. Chapman, M. Charles, Ph. Charpentier, G. Chatzikonstantinidis, M. Chefdeville, V. Chekalina, C. Chen, S. Chen, S.-G. Chitic, V. Chobanova, M. Chrzaszcz, A. Chubykin, P. Ciambrone, X. CidVidal, G. Ciezarek, P. E. L. Clarke, M. Clemencic, H. V. Cliff, J. Closier, V. Coco, J. A. B. Coelho, J. Cogan, E. Cogneras, L. Cojocariu, P. Collins, T. Colombo, A. Comerma-Montells, A. Contu, G. Coombs, S. Coquereau, G. Corti, M. Corvo, C. M. Costa Sobral, B. Couturier, G. A. Cowan, D. C. Craik, A. Crocombe, M. Cruz Torres, R. Currie, C. D’Ambrosio, F. Da Cunha Marinho, C. L. Da Silva, E. Dall’Occo, J. Dalseno, A. Danilina, A. Davis, O. De Aguiar Francisco, K. De Bruyn, S. De Capua, M. De Cian, J. M. De Miranda, L. De Paula, M. De Serio, P. De Simone, C. T. Dean, D. Decamp, L. Del Buono, B. Delaney, H.-P. Dembinski, M. Demmer, A. Dendek, D. Derkach, O. Deschamps, F. Desse, F. Dettori, B. Dey, A. Di Canto, P. Di Nezza, S. Didenko, H. Dijkstra, F. Dordei, M. Dorigo, A. Dosil Suárez, L. Douglas, A. Dovbnya, K. Dreimanis, L. Dufour, G. Dujany, P. Durante, J. M. Durham, D. Dutta, R. Dzhelyadin, M. Dziewiecki, A. Dziurda, A. Dzyuba, S. Easo, U. Egede, V. Egorychev, S. Eidelman, S. Eisenhardt, U. Eitschberger, R. Ekelhof, L. Eklund, S. Ely, A. Ene, S. Escher, S. Esen, T. Evans, A. Falabella, N. Farley, S. Farry, D. Fazzini, L. Federici, P. Fernandez Declara, A. Fernandez Prieto, F. Ferrari, L. Ferreira Lopes, F. Ferreira Rodrigues, M. Ferro-Luzzi, S. Filippov, R. A. Fini, M. Fiorini, M. Firlej, C. Fitzpatrick, T. Fiutowski, F. Fleuret, M. Fontana, F. Fontanelli, R. Forty, V. Franco Lima, M. Frank, C. Frei, J. Fu, W. Funk, C. Färber, M. Féo Pereira Rivello Carvalho, E. Gabriel, A. Gallas Torreira, D. Galli, S. Gallorini, S. Gambetta, Y. Gan, M. Gandelman, P. Gandini, Y. Gao, L. M. Garcia Martin, B. Garcia Plana, J. García Pardiñas, J. Garra Tico, L. Garrido, D. Gascon, C. Gaspar, L. Gavardi, G. Gazzoni, D. Gerick, E. Gersabeck, M. Gersabeck, T. Gershon, D. Gerstel, Ph. Ghez, S. Gianì, V. Gibson, O. G. Girard, P. Gironella Gironell, L. Giubega, K. Gizdov, V. V. Gligorov, D. Golubkov, A. Golutvin, A. Gomes, I. V. Gorelov, C. Gotti, E. Govorkova, J. P. Grabowski, R. Graciani Diaz, L. A. Granado Cardoso, E. Graugés, E. Graverini, G. Graziani, A. Grecu, R. Greim, P. Griffith, L. Grillo, L. Gruber, B. R. Gruberg Cazon, O. Grünberg, C. Gu, E. Gushchin, A. Guth, Yu. Guz, T. Gys, C. Göbel, T. Hadavizadeh, C. Hadjivasiliou, G. Haefeli, C. Haen, S. C. Haines, B. Hamilton, X. Han, T. H. Hancock, S. Hansmann-Menzemer, N. Harnew, S. T. Harnew, T. Harrison, C. Hasse, M. Hatch, J. He, M. Hecker, K. Heinicke, A. Heister, K. Hennessy, L. Henry, E. van Herwijnen, J. Heuel, M. Heß, A. Hicheur, R. Hidalgo Charman, D. Hill, M. Hilton, P. H. Hopchev, W. Hu, W. Huang, Z. C. Huard, W. Hulsbergen, T. Humair, M. Hushchyn, D. Hutchcroft, D. Hynds, P. Ibis, M. Idzik, P. Ilten, K. Ivshin, R. Jacobsson, J. Jalocha, E. Jans, A. Jawahery, F. Jiang, M. John, D. Johnson, C. R. Jones, C. Joram, B. Jost, N. Jurik, S. Kandybei, M. Karacson, J. M. Kariuki, S. Karodia, N. Kazeev, M. Kecke, F. Keizer, M. Kelsey, M. Kenzie, T. Ketel, E. Khairullin, B. Khanji, C. Khurewathanakul, K. E. Kim, T. Kirn, S. Klaver, K. Klimaszewski, T. Klimkovich, S. Koliiev, M. Kolpin, R. Kopecna, P. Koppenburg, I. Kostiuk, S. Kotriakhova, M. Kozeiha, L. Kravchuk, M. Kreps, F. Kress, P. Krokovny, W. Krupa, W. Krzemien, W. Kucewicz, M. Kucharczyk, V. Kudryavtsev, A. K. Kuonen, T. Kvaratskheliya, D. Lacarrere, G. Lafferty, A. Lai, D. Lancierini, G. Lanfranchi, C. Langenbruch, T. Latham, C. Lazzeroni, R. Le Gac, A. Leflat, J. Lefrançois, R. Lefèvre, F. Lemaitre, O. Leroy, T. Lesiak, B. Leverington, P.-R. Li, T. Li, Y. Li, Z. Li, X. Liang, T. Likhomanenko, R. Lindner, F. Lionetto, V. Lisovskyi, G. Liu, X. Liu, D. Loh, A. Loi, I. Longstaff, J. H. Lopes, G. H. Lovell, D. Lucchesi, M. Lucio Martinez, A. Lupato, E. Luppi, O. Lupton, A. Lusiani, X. Lyu, F. Machefert, F. Maciuc, V. Macko, P. Mackowiak, S. Maddrell-Mander, O. Maev, K. Maguire, D. Maisuzenko, M. W. Majewski, S. Malde, B. Malecki, A. Malinin, T. Maltsev, G. Manca, G. Mancinelli, D. Marangotto, J. Maratas, J. F. Marchand, U. Marconi, C. MarinBenito, M. Marinangeli, P. Marino, J. Marks, P. J. Marshall, G. Martellotti, M. Martin, M. Martinelli, D. Martinez Santos, F. Martinez Vidal, A. Massafferri, M. Materok, R. Matev, A. Mathad, Z. Mathe, C. Matteuzzi, A. Mauri, E. Maurice, B. Maurin, A. Mazurov, M. McCann, A. McNab, R. McNulty, J. V. Mead, B. Meadows, C. Meaux, N. Meinert, D. Melnychuk, M. Merk, A. Merli, E. Michielin, D. A. Milanes, E. Millard, M.-N. Minard, L. Minzoni, D. S. Mitzel, A. Mogini, R. D. Moise, J. Molina Rodriguez, T. Mombächer, I. A. Monroy, S. Monteil, M. Morandin, G. Morello, M. J. Morello, O. Morgunova, J. Moron, A. B. Morris, R. Mountain, F. Muheim, M. Mulder, C. H. Murphy, D. Murray, A. Mödden, D. Müller, J. Müller, K. Müller, V. Müller, P. Naik, T. Nakada, R. Nandakumar, A. Nandi, T. Nanut, I. Nasteva, M. Needham, N. Neri, S. Neubert, N. Neufeld, M. Neuner, R. Newcombe, T. D. Nguyen, C. Nguyen-Mau, S. Nieswand, R. Niet, N. Nikitin, A. Nogay, N. S. Nolte, D. P. O’Hanlon, A. Oblakowska-Mucha, V. Obraztsov, S. Ogilvy, R. Oldeman, C. J. G. Onderwater, A. Ossowska, J. M. Otalora Goicochea, P. Owen, A. Oyanguren, P. R. Pais, T. Pajero, A. Palano, M. Palutan, G. Panshin, A. Papanestis, M. Pappagallo, L. L. Pappalardo, W. Parker, C. Parkes, G. Passaleva, A. Pastore, M. Patel, C. Patrignani, A. Pearce, A. Pellegrino, G. Penso, M. Pepe Altarelli, S. Perazzini, D. Pereima, P. Perret, L. Pescatore, K. Petridis, A. Petrolini, A. Petrov, S. Petrucci, M. Petruzzo, B. Pietrzyk, G. Pietrzyk, M. Pikies, M. Pili, D. Pinci, J. Pinzino, F. Pisani, A. Piucci, V. Placinta, S. Playfer, J. Plews, M. Plo Casasus, F. Polci, M. Poli Lener, A. Poluektov, N. Polukhina, I. Polyakov, E. Polycarpo, G. J. Pomery, S. Ponce, A. Popov, D. Popov, S. Poslavskii, C. Potterat, E. Price, J. Prisciandaro, C. Prouve, V. Pugatch, A. Puig Navarro, H. Pullen, G. Punzi, W. Qian, J. Qin, R. Quagliani, B. Quintana, B. Rachwal, J. H. Rademacker, M. Rama, M. RamosPernas, M. S. Rangel, F. Ratnikov, G. Raven, M. Ravonel Salzgeber, M. Reboud, F. Redi, S. Reichert, A. C. dos Reis, F. Reiss, C. Remon Alepuz, Z. Ren, V. Renaudin, S. Ricciardi, S. Richards, K. Rinnert, P. Robbe, A. Robert, A. B. Rodrigues, E. Rodrigues, J. A. Rodriguez Lopez, M. Roehrken, S. Roiser, A. Rollings, V. Romanovskiy, A. Romero Vidal, M. Rotondo, M. S. Rudolph, T. Ruf, J. Ruiz Vidal, J. J. Saborido Silva, N. Sagidova, B. Saitta, V. Salustino Guimaraes, C. Sanchez Gras, C. Sanchez Mayordomo, B. Sanmartin Sedes, R. Santacesaria, C. Santamarina Rios, M. Santimaria, E. Santovetti, G. Sarpis, A. Sarti, C. Satriano, A. Satta, M. Saur, D. Savrina, S. Schael, M. Schellenberg, M. Schiller, H. Schindler, M. Schmelling, T. Schmelzer, B. Schmidt, O. Schneider, A. Schopper, H. F. Schreiner, M. Schubiger, M. H. Schune, R. Schwemmer, B. Sciascia, A. Sciubba, A. Semennikov, E. S. Sepulveda, A. Sergi, N. Serra, J. Serrano, L. Sestini, A. Seuthe, P. Seyfert, M. Shapkin, Y. Shcheglov, T. Shears, L. Shekhtman, V. Shevchenko, E. Shmanin, B. G. Siddi, R. Silva Coutinho, L. Silva de Oliveira, G. Simi, S. Simone, I. Skiba, N. Skidmore, T. Skwarnicki, M. W. Slater, J. G. Smeaton, E. Smith, I. T. Smith, M. Smith, M. Soares, l. Soares Lavra, M. D. Sokoloff, F. J. P. Soler, B. Souza De Paula, B. Spaan, E. Spadaro Norella, P. Spradlin, F. Stagni, M. Stahl, S. Stahl, P. Stefko, S. Stefkova, O. Steinkamp, S. Stemmle, O. Stenyakin, M. Stepanova, H. Stevens, A. Stocchi, S. Stone, B. Storaci, S. Stracka, M. E. Stramaglia, M. Straticiuc, U. Straumann, S. Strokov, J. Sun, L. Sun, K. Swientek, A. Szabelski, T. Szumlak, M. Szymanski, S. T’Jampens, Z. Tang, A. Tayduganov, T. Tekampe, G. Tellarini, F. Teubert, E. Thomas, J. van Tilburg, M. J. Tilley, V. Tisserand, M. Tobin, S. Tolk, L. Tomassetti, D. Tonelli, D. Y. Tou, R. Tourinho Jadallah Aoude, E. Tournefier, M. Traill, M. T. Tran, A. Trisovic, A. Tsaregorodtsev, G. Tuci, A. Tully, N. Tuning, A. Ukleja, A. Usachov, A. Ustyuzhanin, U. Uwer, A. Vagner, V. Vagnoni, A. Valassi, S. Valat, G. Valenti, R. Vazquez Gomez, P. Vazquez Regueiro, S. Vecchi, M. van Veghel, J. J. Velthuis, M. Veltri, G. Veneziano, A. Venkateswaran, M. Vernet, M. Veronesi, N. V. Veronika, M. Vesterinen, J. V. Viana Barbosa, D. Vieira, M. Vieites Diaz, H. Viemann, X. Vilasis-Cardona, A. Vitkovskiy, M. Vitti, V. Volkov, A. Vollhardt, D. Vom Bruch, B. Voneki, A. Vorobyev, V. Vorobyev, J. A. de Vries, C. Vázquez Sierra, R. Waldi, J. Walsh, J. Wang, M. Wang, Y. Wang, Z. Wang, D. R. Ward, H. M. Wark, N. K. Watson, D. Websdale, A. Weiden, C. Weisser, M. Whitehead, J. Wicht, G. Wilkinson, M. Wilkinson, I. Williams, M. R. J. Williams, M. Williams, T. Williams, F. F. Wilson, M. Winn, J. Wishahi, W. Wislicki, M. Witek, G. Wormser, S. A. Wotton, K. Wyllie, D. Xiao, Y. Xie, A. Xu, M. Xu, Q. Xu, Z. Xu, Z. Xu, Z. Yang, Z. Yang, Y. Yao, L. E. Yeomans, H. Yin, J. Yu, X. Yuan, O. Yushchenko, K. A. Zarebski, M. Zavertyaev, D. Zhang, L. Zhang, W. C. Zhang, Y. Zhang, A. Zhelezov, Y. Zheng, X. Zhu, V. Zhukov, J. B. Zonneveld, S. Zucchelli

**Affiliations:** 1 Centro Brasileiro de Pesquisas Físicas (CBPF), Rio de Janeiro, Brazil; 2 Universidade Federal do Rio de Janeiro (UFRJ), Rio de Janeiro, Brazil; 3 Center for High Energy Physics, Tsinghua University, Beijing, China; 4 Institute Of High Energy Physics (IHEP), Beijing, China; 5 Univ. Grenoble Alpes, Univ. Savoie Mont Blanc, CNRS, IN2P3-LAPP, Annecy, France; 6 Clermont Université, Université Blaise Pascal, CNRS/IN2P3, LPC, Clermont-Ferrand, France; 7 Aix-Marseille Univ, CNRS/IN2P3, CPPM, Marseille, France; 8 LAL, Univ. Paris-Sud, CNRS/IN2P3, Université Paris-Saclay, Orsay, France; 9 LPNHE, Sorbonne Université, Paris Diderot Sorbonne Paris Cité, CNRS/IN2P3, Paris, France; 10 I. Physikalisches Institut, RWTH Aachen University, Aachen, Germany; 11 Fakultät Physik, Technische Universität Dortmund, Dortmund, Germany; 12 Max-Planck-Institut für Kernphysik (MPIK), Heidelberg, Germany; 13 Physikalisches Institut, Ruprecht-Karls-Universität Heidelberg, Heidelberg, Germany; 14 School of Physics, University College Dublin, Dublin, Ireland; 15 INFN Sezione di Bari, Bari, Italy; 16 INFN Sezione di Bologna, Bologna, Italy; 17 INFN Sezione di Ferrara, Ferrara, Italy; 18 INFN Sezione di Firenze, Firenze, Italy; 19 INFN Laboratori Nazionali di Frascati, Frascati, Italy; 20 INFN Sezione di Genova, Genoa, Italy; 21 INFN Sezione di Milano-Bicocca, Milan, Italy; 22 INFN Sezione di Milano, Milan, Italy; 23 INFN Sezione di Cagliari, Monserrato, Italy; 24 INFN Sezione di Padova, Padua, Italy; 25 INFN Sezione di Pisa, Pisa, Italy; 26 INFN Sezione di Roma Tor Vergata, Rome, Italy; 27 INFN Sezione di Roma La Sapienza, Rome, Italy; 28 Nikhef National Institute for Subatomic Physics, Amsterdam, The Netherlands; 29 Nikhef National Institute for Subatomic Physics and VU University Amsterdam, Amsterdam, The Netherlands; 30 Henryk Niewodniczanski Institute of Nuclear Physics Polish Academy of Sciences, Kraków, Poland; 31 Faculty of Physics and Applied Computer Science, AGH-University of Science and Technology, Kraków, Poland; 32 National Center for Nuclear Research (NCBJ), Warsaw, Poland; 33 Horia Hulubei National Institute of Physics and Nuclear Engineering, Bucharest-Magurele, Romania; 34 Petersburg Nuclear Physics Institute (PNPI), Gatchina, Russia; 35 Institute of Theoretical and Experimental Physics (ITEP), Moscow, Russia; 36 Institute of Nuclear Physics, Moscow State University (SINP MSU), Moscow, Russia; 37 Institute for Nuclear Research of the Russian Academy of Sciences (INR RAS), Moscow, Russia; 38 Yandex School of Data Analysis, Moscow, Russia; 39 Budker Institute of Nuclear Physics (SB RAS), Novosibirsk, Russia; 40 Institute for High Energy Physics (IHEP), Protvino, Russia; 41 ICCUB, Universitat de Barcelona, Barcelona, Spain; 42 Instituto Galego de Física de Altas Enerxías (IGFAE), Universidade de Santiago de Compostela, Santiago de Compostela, Spain; 43 European Organization for Nuclear Research (CERN), Geneva, Switzerland; 44 Institute of Physics, Ecole Polytechnique Fédérale de Lausanne (EPFL), Lausanne, Switzerland; 45 Physik-Institut, Universität Zürich, Zürich, Switzerland; 46 NSC Kharkiv Institute of Physics and Technology (NSC KIPT), Kharkiv, Ukraine; 47 Institute for Nuclear Research of the National Academy of Sciences (KINR), Kyiv, Ukraine; 48 University of Birmingham, Birmingham, UK; 49 H.H. Wills Physics Laboratory, University of Bristol, Bristol, UK; 50 Cavendish Laboratory, University of Cambridge, Cambridge, UK; 51 Department of Physics, University of Warwick, Coventry, UK; 52 STFC Rutherford Appleton Laboratory, Didcot, UK; 53 School of Physics and Astronomy, University of Edinburgh, Edinburgh, UK; 54 School of Physics and Astronomy, University of Glasgow, Glasgow, UK; 55 Oliver Lodge Laboratory, University of Liverpool, Liverpool, UK; 56 Imperial College London, London, UK; 57 School of Physics and Astronomy, University of Manchester, Manchester, UK; 58 Department of Physics, University of Oxford, Oxford, UK; 59 Massachusetts Institute of Technology, Cambridge, MA USA; 60 University of Cincinnati, Cincinnati, OH USA; 61 University of Maryland, College Park, MD USA; 62 Syracuse University, Syracuse, NY USA; 63 Laboratory of Mathematical and Subatomic Physics, Constantine, Algeria; 64 Pontifícia Universidade Católica do Rio de Janeiro (PUC-Rio), Rio de Janeiro, Brazil; 65 University of Chinese Academy of Sciences, Beijing, China; 66 South China Normal University, Guangzhou, China; 67 School of Physics and Technology, Wuhan University, Wuhan, China; 68 Institute of Particle Physics, Central China Normal University, Wuhan, Hubei China; 69 Departamento de Fisica, Universidad Nacional de Colombia, Bogotá, Colombia; 70 Institut für Physik, Universität Rostock, Rostock, Germany; 71 Van Swinderen Institute, University of Groningen, Groningen, Netherlands; 72 National Research Centre Kurchatov Institute, Moscow, Russia; 73 National University of Science and Technology “MISIS”, Moscow, Russia; 74 National Research Tomsk Polytechnic University, Tomsk, Russia; 75 Instituto de Fisica Corpuscular, Centro Mixto Universidad de Valencia-CSIC, Valencia, Spain; 76 University of Michigan, Ann Arbor, USA; 77 Los Alamos National Laboratory (LANL), Los Alamos, USA

## Abstract

A Dalitz plot analysis of ${{B} ^0} \!\rightarrow \eta _c(1S) {{K} ^+} {{\pi } ^-}$ decays is performed using data samples of *pp* collisions collected with the $\text{ LHCb }$ detector at centre-of-mass energies of ${\sqrt{s}} =7,~8$ and $13{\,\mathrm {Te}\mathrm {V}}$, corresponding to a total integrated luminosity of $4.7 \,\text{ fb }^{-1}$. A satisfactory description of the data is obtained when including a contribution representing an exotic $\eta _c(1S) \pi ^-$ resonant state. The significance of this exotic resonance is more than three standard deviations, while its mass and width are $4096 \pm 20~^{+18}_{-22} \,\mathrm {Me}\mathrm {V}$ and $152 \pm 58~^{+60}_{-35} \,\mathrm {Me}\mathrm {V}$, respectively. The spin-parity assignments $J^P=0^+$ and $J^{P}=1^-$ are both consistent with the data. In addition, the first measurement of the ${{B} ^0} \!\rightarrow \eta _c(1S) {{K} ^+} {{\pi } ^-}$ branching fraction is performed and gives

$$\begin{aligned} \displaystyle \mathcal {B}({{B} ^0} \!\rightarrow \eta _c(1S) {{K} ^+} {{\pi } ^-} ) = (5.73 \pm 0.24 \pm 0.13 \pm 0.66) \times 10^{-4}, \end{aligned}$$

where the first uncertainty is statistical, the second systematic, and the third is due to limited knowledge of external branching fractions.

## Introduction

Since the discovery of the *X*(3872) state in 2003 [1], several exotic hadron candidates have been observed, as reported in recent reviews [2–7].^1^ The decay modes of these states indicate that they must contain a heavy quark–antiquark pair in their internal structure; however, they cannot easily be accommodated as an unassigned charmonium or bottomonium state due to either their mass, decay properties or electric charge, which are inconsistent with those of pure charmonium or bottomonium states. Different interpretations have been proposed about their nature [2–4], including their quark composition and binding mechanisms. In order to improve the understanding of these hadrons, it is important to search for new exotic candidates, along with new production mechanisms and decay modes of already observed unconventional states.

**Fig. 1:**
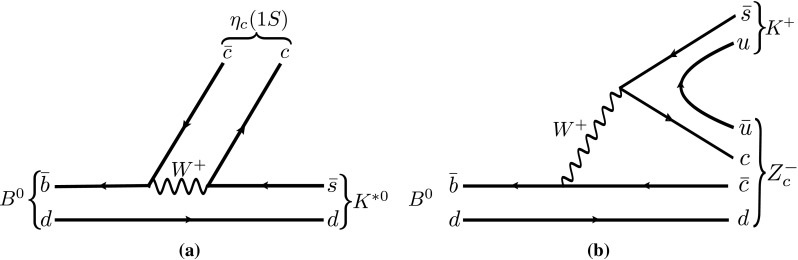
Feynman diagrams for **a** $B^0 \rightarrow \eta _cK^{*0}$ and **b** $B^0 \rightarrow Z_c^-K^+$ decay sequences

The $Z_c(3900)^-$ state, discovered by the BESIII collaboration in the ${{J /\psi }} \pi ^-$ final state [9], and confirmed by the Belle  [10] and CLEO  [11] collaborations, can be interpreted as a hadrocharmonium state, where the compact heavy quark–antiquark pair interacts with the surrounding light quark mesonic excitation by a QCD analogue of the van der Waals force [12]. This interpretation of the $Z_c(3900)^-$ state predicts an as-yet-unobserved charged charmonium-like state with a mass of approximately $\,\mathrm {[} 3800]{MeV}$ whose dominant decay mode is to the ${\eta _{c}} {{\pi } ^-}$ system.^2^ Alternatively, states like the $Z_c(3900)^-$ meson could be interpreted as analogues of quarkonium hybrids, where the excitation of the gluon field (the valence gluon) is replaced by an isospin-1 excitation of the gluon and light-quark fields [13]. This interpretation, which is based on lattice QCD, predicts different multiplets of charmonium tetraquarks, comprising states with quantum numbers allowing the decay into the ${\eta _{c}} {{\pi } ^-}$ system. The ${\eta _{c}} {{\pi } ^-}$ system carries isospin $I=1$, *G*-parity $G=-1$, spin $J=L$ and parity $P=(-1)^{L}$, where *L* is the orbital angular momentum between the $\eta _{c}$ and the ${\pi } ^-$ mesons. Lattice QCD calculations [14, 15] predict the mass and quantum numbers of these states, comprising a $I^G(J^P)=1^-(0^+)$ state of mass $\,\mathrm {[} 4025 \pm 49]{MeV}$, a $I^G(J^P)=1^-(1^-)$ state of mass $\,\mathrm {[} 3770 \pm 42]{MeV}$, and a $I^G(J^P)=1^-(2^+)$ state of mass $\,\mathrm {[} 4045 \pm 44]{MeV}$. The $Z_c(4430)^-$ resonance, discovered by the Belle collaboration [16] and confirmed by LHCb  [17, 18], could also fit into this scenario. Another prediction of a possible exotic candidate decaying to the ${\eta _{c}} {{\pi } ^-}$ system is provided by the diquark model [19], where quarks and diquarks are the fundamental units to build a rich spectrum of hadrons, including the exotic states observed thus far. The diquark model predicts a $J^P=0^+$ candidate below the open-charm threshold that could decay into the ${\eta _{c}} {{\pi } ^-}$ final state. Therefore, the discovery of a charged charmonium-like meson in the ${\eta _{c}} {{\pi } ^-}$ system would provide important input towards understanding the nature of exotic hadrons.

In this article, the ${{{B} ^0} \!\rightarrow {\eta _{c}} {{K} ^+} {{\pi } ^-} }$ decay is studied for the first time, with the $\eta _{c}$ meson reconstructed using the ${p} {\overline{{p}}}$ decay mode. The decay is expected to proceed through ${{K} ^{*0}} \!\rightarrow {{K} ^+} {{\pi } ^-}$ intermediate states, where ${K} ^{*0}$ refers to any neutral kaon resonance, following the diagram shown in Fig. 1a. If the decay also proceeds through exotic resonances in the ${\eta _{c}} {{\pi } ^-}$system, denoted by $Z_c^-$ states in the following, a diagram like that shown in Fig. 1b would contribute. The ${{{B} ^0} \!\rightarrow {\eta _{c}} {{K} ^+} {{\pi } ^-} }$ decay involves only pseudoscalar mesons, hence it is fully described by two independent kinematic quantities. Therefore, the Dalitz plot (DP) analysis technique [20] can be used to completely characterise the decay.

The data sample used corresponds to an integrated luminosity of $$4.7 \,\text{ fb }^{-1} $$ of $${p} {p} $$ collision data collected with the LHCb detector at centre-of-mass energies of $${\sqrt{s}} =7,~8$$ and $$13{\,\mathrm {Te}\mathrm {V}} $$ in 2011, 2012 and 2016, respectively. Data collected in 2011 and 2012 are referred to as Run 1 data, while data collected in 2016 are referred to as Run 2 data.

This paper is organised as follows. A brief description of the LHCb detector as well as the reconstruction and simulation software is given in Sect. 2. The selection of  candidates is described in Sect. 3, and the first measurement of the ${{{B} ^0} \!\rightarrow {\eta _{c}} {{K} ^+} {{\pi } ^-} }$ branching fraction is presented in Sect. 4. An overview of the DP analysis formalism is given in Sect. 5. Details of the implementation of the DP fit are presented in Sect. 6. The evaluation of systematic uncertainties is given in Sect. 7. The results are summarised in Sect. 8.

## Detector and simulation

The LHCb detector [21, 22] is a single-arm forward spectrometer covering the pseudorapidity range $$2<\eta <5$$, designed for the study of particles containing $$b $$ or $$c $$ quarks. The detector includes a high-precision tracking system consisting of a silicon-strip vertex detector surrounding the *pp* interaction region, a large-area silicon-strip detector located upstream of a dipole magnet with a bending power of about $$4{\mathrm {\,Tm}}$$, and three stations of silicon-strip detectors and straw drift tubes placed downstream of the magnet. The tracking system provides a measurement of the momentum, $$p$$, of charged particles with a relative uncertainty that varies from 0.5% at low momentum to 1.0% at 200$$\,\mathrm {Ge}\mathrm {V}$$. The minimum distance of a track to a primary vertex (PV), the impact parameter, is measured with a resolution of $$(15+29/p_{\mathrm { T}}){\,\upmu \mathrm {m}} $$, where $$p_{\mathrm { T}}$$ is the component of the momentum transverse to the beam, in $$\,\mathrm {Ge}\mathrm {V}$$. Different types of charged hadrons are distinguished using information from two ring-imaging Cherenkov (RICH) detectors. Photons, electrons and hadrons are identified by a calorimeter system consisting of scintillating-pad and preshower detectors, an electromagnetic calorimeter and a hadronic calorimeter. Muons are identified by a system composed of alternating layers of iron and multiwire proportional chambers.

The online event selection is performed by a trigger [23], which consists of a hardware stage, based on information from the calorimeter and muon systems, followed by a software stage, which applies a full event reconstruction. At the hardware trigger stage, events are required to have a hadron with high transverse energy in the calorimeters. The software trigger requires a two-, three- or four-tracks secondary vertex with a significant displacement from any PV. At least one charged particle must have a large transverse momentum and be inconsistent with originating from a PV. A multivariate algorithm [24, 25] is used to identify secondary vertices that are consistent with *b*-hadron decays.

Simulated events, generated uniformly in the phase space of the  or ${{{B} ^0} \!\rightarrow {\eta _{c}} {{K} ^+} {{\pi } ^-} }$ decay modes, are used to develop the selection, to validate the fit models and to evaluate the efficiencies entering the branching fraction measurement and the DP analysis. In the simulation, *pp* collisions are generated using Pythia  [26, 27] with a specific LHCb configuration [28]. Decays of hadronic particles are described by EvtGen  [29], in which final-state radiation is generated using Photos  [30]. The interaction of the generated particles with the detector, and its response, are implemented using the Geant4 toolkit [31, 32] as described in Ref. [33].

## Selection

An initial offline selection comprising loose criteria is applied to reconstructed particles, where the associated trigger decision was due to the ${B} ^0$ candidate. The final-state tracks are required to have $p > \,\mathrm {[} 1500]{MeV}$, $p_{\mathrm { T}} > \,\mathrm {[} 300]{MeV}$, and to be inconsistent with originating from any PV in the event. Loose particle identification (PID) criteria are applied, requiring the particles to be consistent with either the proton, kaon or pion hypothesis. All tracks are required to be within the acceptance of the RICH detectors ($2.0< \eta < 4.9$). Moreover, protons and antiprotons are required to have momenta larger than $\,\mathrm {[} 8]{GeV}$ ($\,\mathrm {[} 11]{GeV}$) to avoid kinematic regions where proton-kaon separation is limited for Run 1 (Run 2) data.

The $${{B} ^0} $$ candidates are required to have a small $$\chi ^2_{\text {IP}} $$ with respect to a PV, where $$\chi ^2_{\text {IP}} $$ is defined as the difference in the vertex-fit $$\chi ^2 $$ of a given PV reconstructed with and without the candidate under consideration. The PV providing the smallest $$\chi ^2_{\text {IP}} $$ value is associated to the $${{B} ^0} $$ candidate. The $${{B} ^0} $$ candidate is required to be consistent with originating from this PV by applying a criterion on the direction angle (DIRA) between the $${{B} ^0} $$ candidate momentum vector and the distance vector between the PV to the $${{B} ^0} $$ decay vertex. When building the $${{B} ^0} $$ candidates, the resolution on kinematic quantities such as the $$m({p} {\overline{{p}}})$$ distribution, and the Dalitz variables that will be defined in Sect. 5, is improved by performing a kinematic fit [34] in which the $${{B} ^0} $$ candidate is constrained to originate from its associated PV, and its reconstructed invariant mass is constrained to the known $${{B} ^0} $$ mass [8].

A boosted decision tree (BDT) [35, 36] algorithm is used to further suppress the combinatorial background that arises when unrelated particles are combined to form a $${{B} ^0} $$ candidate. The training of the BDT is performed using simulated  decays as the signal sample and candidates from the high-mass data sideband as the background sample, defined as the  range. The input variables to the BDT classifiers are the same for Run 1 and 2 samples and comprise typical discriminating variables of *b*-hadron decays: the vertex-fit $$\chi ^2_{\text {vtx}} $$, $$\chi ^2_{\text {IP}} $$, DIRA and flight distance significance of the reconstructed $${{B} ^0} $$ candidates; the maximum distance of closest approach between final-state particles; and the maximum and minimum *p* and $$p_{\mathrm { T}} $$ of the proton and antiproton.

The requirements placed on the output of the BDT algorithm and PID variables are simultaneously optimised to maximise the figure of merit defined as . Here *S* is the observed  yield before any BDT selection multiplied by the efficiency of the BDT requirement evaluated using simulated decays, while *B* is the the combinatorial background yield. The training of the BDT and the optimisation of the selection are performed separately for Run 1 and 2 data to accommodate for differences in the two data-taking periods.

## Branching fraction measurement

The measurement of the ${{{B} ^0} \!\rightarrow {\eta _{c}} {{K} ^+} {{\pi } ^-} }$ branching fraction is performed relative to that of the ${{B} ^0} \!\rightarrow {{J /\psi }} {{K} ^+} {{\pi } ^-}$ normalisation channel, where the ${{J /\psi }}$ meson is also reconstructed in the ${p} {\overline{{p}}}$ decay mode. A two-stage fit procedure to the combined Run 1 and 2 data sample is used. In the first stage, an extended unbinned maximum-likelihood (UML) fit is performed to the  distribution in order to separate the  and background contributions. The RooFit package [37] is used to perform the fit, and the *sPlot* technique [38] is applied to assign weights for each candidate to subtract the background contributions. In the second stage, a weighted UML fit to the ${p} {\overline{{p}}}$ invariant-mass spectrum is performed to disentangle the ${\eta _{c}}, {{J /\psi }}$, and nonresonant (NR) contributions. The efficiency-corrected yield ratio is1where  and  are the observed  and  yields, while  and  are the total efficiencies, which are obtained from a combination of simulated and calibration samples. The  branching fraction is determined as2where $\mathcal {B}({{B} ^0} \!\rightarrow {{J /\psi }} {{K} ^+} {{\pi } ^-} ) = (1.15 \pm 0.05) \times 10^{-3}$, $\mathcal {B}({{J /\psi }} \!\rightarrow {p} {\overline{{p}}} ) = (2.121 \pm 0.029) \times 10^{-3}$ and  are the external branching fractions taken from Ref. [8].

### Signal and normalisation yields

The first-stage UML fit to the  distribution is performed in the $5180-5430\,\mathrm {Me}\mathrm {V}$ range. The  signal decays, ${{B} ^0_{s}} \!\rightarrow {p} {\overline{{p}}} {{K} ^+} {{\pi } ^-}$ decays and various categories of background are present in this range. In addition to the combinatorial background, partially reconstructed backgrounds are present originating from *b*-hadron decays with additional particles that are not part of the reconstructed decay chain, such as a ${\pi } ^0$ meson or a photon. Another source of background is *b*-hadron decays where one of the final-state particles has been incorrectly identified, which includes the decays ${{B} ^0} \!\rightarrow {p} {\overline{{p}}} {{\pi } ^+} {{\pi } ^-}$ and ${{B} ^0_{s}} \!\rightarrow {p} {\overline{{p}}} {{K} ^+} {{K} ^-}$. The ${{\overline{D}{}} {}^0} \!\rightarrow {{K} ^+} {{\pi } ^-}$ and ${{\overline{\Lambda }} {}^-_{c}} \!\rightarrow {\overline{{p}}} {{K} ^+} {{\pi } ^-}$ decays are removed by excluding the mass range $1845-1885\,\mathrm {Me}\mathrm {V}$ in the $m({{K} ^+} {{\pi } ^-})$ distribution and the range $2236-2336\,\mathrm {Me}\mathrm {V}$ in the $m({\overline{{p}}} {{K} ^+} {{\pi } ^-})$ distribution, respectively. The latter veto also removes partially reconstructed *b*-hadron decays.

**Fig. 2:**
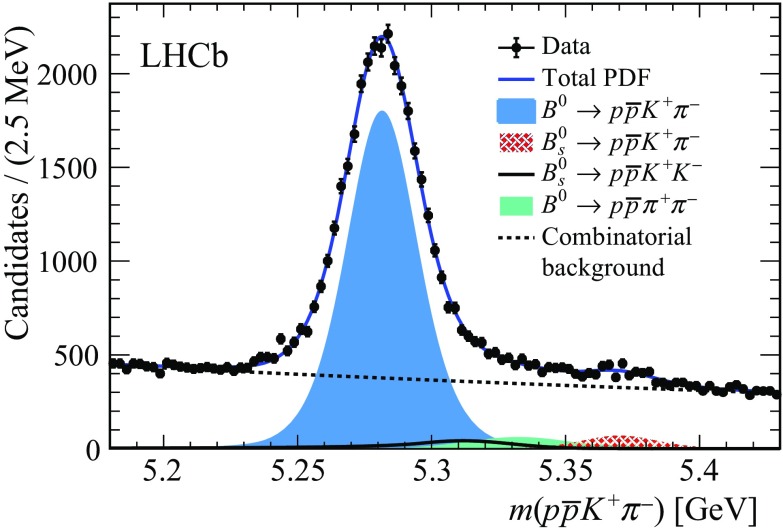
Distribution of the ${p} {\overline{{p}}} {{K} ^+} {{\pi } ^-}$ invariant mass. The solid blue curve is the projection of the total fit result. The components are shown in the legend

Both the  and $${{B} ^0_{s}} \!\rightarrow {p} {\overline{{p}}} {{K} ^+} {{\pi } ^-} $$ components are modelled by Hypatia functions [39]. The Hypatia distribution is a generalisation of the Crystall Ball function [40], where the Gaussian core of the latter is replaced by a hyperbolic core to take into account the distortion on the measured mass due to different sources of uncertainty. The Hypatia functions share a common resolution parameter, while the tail parameters are fixed to the values obtained from the corresponding simulated sample. The distributions of the misidentified $${{B} ^0} \!\rightarrow {p} {\overline{{p}}} {{\pi } ^+} {{\pi } ^-} $$ and $${{B} ^0_{s}} \!\rightarrow {p} {\overline{{p}}} {{K} ^+} {{K} ^-} $$ backgrounds are described by Crystal Ball functions, with parameters fixed to the values obtained from simulation. The combinatorial background is modelled using an exponential function. The masses of the $${B} ^0$$ and $${B} ^0_{s} $$ mesons, the resolution parameter of the Hypatia functions, the slope of the exponential function, and all the yields, are free to vary in the fit to the data. Using the information from the fit to the  distribution, shown in Fig. 2,  signal weights are computed and the background components are subtracted using the *sPlot* technique [38]. About $$3.0 \times 10^4$$ $${B} ^0$$ decays are observed. Correlations between the $${p} {\overline{{p}}} $$ and $$p $$ $$\overline{{p}}$$ $${K} ^+$$ $${\pi } ^-$$ invariant-mass variables for both signal and background are found to be negligible.

**Fig. 3:**
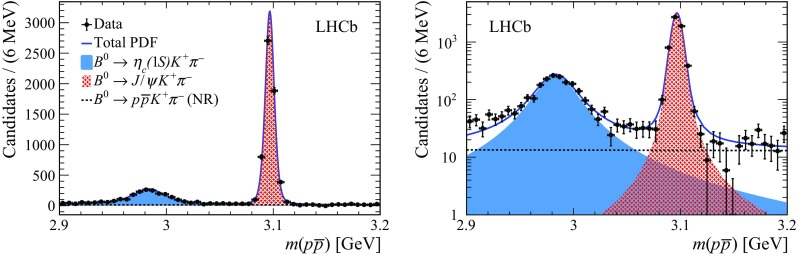
Distribution of the ${p} {\overline{{p}}}$ invariant mass in (left) linear and (right) logarithmic vertical-axis scale for weighted  candidates obtained by using the *sPlot* technique. The solid blue curve is the projection of the total fit result. The full azure, tight-cross-hatched red and dashed-black line areas show the  and NR ${p} {\overline{{p}}}$ contributions, respectively

The second-stage UML fit is then performed to the weighted ${p} {\overline{{p}}}$ invariant-mass distribution in the mass range $2700-3300\,\mathrm {Me}\mathrm {V}$, which includes , and NR  contributions. The ${p} {\overline{{p}}}$ invariant-mass distribution of  candidates is described by the convolution of a nonrelativistic Breit–Wigner function and a Gaussian function describing resolution effects. Using simulated samples, the ${p} {\overline{{p}}}$ invariant-mass resolution is found to be $\approx \,\mathrm {[} 5]{MeV}$. Given the width  [8], the impact of the detector resolution on the  lineshape is small. The ${{J /\psi }}$ resonance, having a small natural width, is parametrised using an Hypatia function, with tail parameters fixed to the values obtained from the corresponding simulated sample. The same resolution parameter is used for the  and ${{J /\psi }}$ contributions, which is free to vary in the fit to the data. The  and ${{J /\psi }}$ masses are also floating, while the  natural width is Gaussian constrained to the known value [8]. The NR  contribution is parametrised with an exponential function with the slope free to vary in the fit. All yields are left unconstrained in the fit. A possible term describing the interference between the  resonance and the NR ${p} {\overline{{p}}}$ S-wave is investigated and found to be negligible. The result of the fit to the weighted ${p} {\overline{{p}}}$ invariant-mass distribution is shown in Fig. 3. The yields of the  and ${{B} ^0} \!\rightarrow {{J /\psi }} {{K} ^+} {{\pi } ^-}$ fit components, entering Eq. (1), are $2105 \pm 75$ and $5899 \pm 86$, respectively.

### Ratio of efficiencies

The ratio of efficiencies of Eq. (1) is obtained from  and ${{B} ^0} \!\rightarrow {{J /\psi }} {{K} ^+} {{\pi } ^-}$ simulated samples, both selected using the same criteria used in data. Since these decays have the same final-state particles and similar kinematic distributions, the ratio of efficiencies is expected to be close to unity. The efficiencies are computed as the product of the geometrical acceptance of the LHCb detector, the reconstruction efficiency and the efficiency of the offline selection criteria, including the trigger and PID requirements. The efficiency of the PID requirements is obtained using calibration samples of pions, kaons and protons, as a function of the particle momentum, pseudorapidity and the multiplicity of the event, *e.g.* the number of charged particles in the event [41]. The final ratio of efficiencies is given by3which is compatible with unity as expected.

**Table 1:** Relative systematic uncertainties on the ratio *R* of Eq. (1). The total systematic uncertainty is obtained from the quadratic sum of the individual sources

Source	Systematic uncertainty (%)
Fixed shape parameters	0.8
Resolution model	0.3
NR $p\bar{p}$ model	1.7
Efficiency ratio	1.1
Total	2.2

### Systematic uncertainties

Table 1 summarises the systematic uncertainties on the measurement of the ratio *R* of Eq. (1). Since the kinematic distributions of the signal and normalisation channel are similar, the uncertainties corresponding to the reconstruction and selection efficiencies largely cancel in the ratio of branching fractions. A new value of the ratio *R* is computed for each source of systematic uncertainty, and its difference with the nominal value is taken as the associated systematic uncertainty. The overall systematic uncertainty is assigned by combining all contributions in quadrature.

The systematic uncertainty arising from fixing the shape parameters of the Hypatia functions used to parametrise the ${B} ^0$ and ${{J /\psi }}$ components is evaluated by repeating the fits and varying all shape parameters simultaneously. These shape parameters are varied according to normal distributions, taking into account the correlations between the parameters and with variances related to the size of the simulated samples.

To assign a systematic uncertainty arising from the model used to describe the detector resolution, the fits are repeated for each step replacing the Hypatia functions by Crystal Ball functions, whose parameters are obtained from simulation.

The systematic uncertainty associated to the parametrisation of the NR  contribution is determined by replacing the exponential function with a linear function.

The systematic uncertainty associated to the determination of the efficiency involves contributions arising from the weighting procedure of the calibration samples used to determine the PID efficiencies. The granularity of the binning in the weighting procedure is halved and doubled.

The free shape parameters in the first stage UML fit lead to uncertainties that are not taken into account by the *sPlot* technique. In order to estimate this effect, these parameters are varied within their uncertainties and the signal weights are re-evaluated. The variations on the ratio *R* resulting from the second stage UML fit are found to be negligible.

### Results

The ratio *R* is determined to be

$$\begin{aligned} R = 0.357 \pm 0.015 \pm 0.008, \end{aligned}$$

where the first uncertainty is statistical and the second systematic. The statistical uncertainty includes contributions from the per-candidate weights obtained using the *sPlot* technique. The value of *R* is used to compute the  branching fraction using Eq. (2) which giveswhere the first uncertainty is statistical, the second systematic, and the third is due to the limited knowledge of the external branching fractions.

## Dalitz plot formalism

The phase space for a three-body decay involving only pseudoscalar particles can be represented in a DP, where two of the three possible two-body invariant-mass-squared combinations, here $m^2({{K} ^+} {{\pi } ^-})$ and $m^2({\eta _{c}} {{\pi } ^-})$, are used to define the DP axes. However, given the sizeable natural width of the  meson, the invariant mass $m({p} {\overline{{p}}})$ is used instead of the known value of the  mass [8] to compute the kinematic quantities such as  and the helicity angles.

The isobar model [42–44] is used to write the decay amplitude as a coherent sum of amplitudes from resonant and NR intermediate processes as

4 $$\begin{aligned}&\mathcal {A}[m^2({{K} ^+} {{\pi } ^-}),m^2({\eta _{c}} {{\pi } ^-})] \nonumber \\&\quad = \sum _{j=1}^{N}c_j\mathcal {F}_j[m^2({{K} ^+} {{\pi } ^-}),m^2({\eta _{c}} {{\pi } ^-})], \end{aligned}$$

where $c_j$ are complex coefficients giving the relative contribution of each intermediate process. The $\mathcal {F}_j[m^2({{K} ^+} {{\pi } ^-}),m^2({\eta _{c}} {{\pi } ^-})]$ complex functions describe the resonance dynamics and are normalised such that the integral of their squared magnitude over the DP is unity

5 $$\begin{aligned}&\int _{\text {DP}} |\mathcal {F}_j[m^2({{K} ^+} {{\pi } ^-}),m^2({\eta _{c}} {{\pi } ^-})]|^2 \nonumber \\&\quad \times \text {d}m^2({{K} ^+} {{\pi } ^-})~\text {d}m^2({\eta _{c}} {{\pi } ^-})= 1. \end{aligned}$$

Each $\mathcal {F}_j[m^2({{K} ^+} {{\pi } ^-}),m^2({\eta _{c}} {{\pi } ^-})]$ contribution is composed of the product of several factors. For a ${{K} ^+} {{\pi } ^-}$ resonance, for instance,

6 $$\begin{aligned}&\mathcal {F}[m^2({{K} ^+} {{\pi } ^-}),m^2({\eta _{c}} {{\pi } ^-})] \nonumber \\&\quad = \mathcal {N} \times X(|\vec {p}| r_{\text {BW}}) \times X(|\vec {q}|r_{\text {BW}}) Z(\vec {p},\vec {q})\nonumber \\&\qquad \times T[m({{K} ^+} {{\pi } ^-})], \end{aligned}$$

where $\displaystyle \mathcal {N}$ is a normalisation constant and $\vec {p}$ and $\vec {q}$ are the momentum of the accompanying particle (the  meson in this case) and the momentum of one of the resonance decay products, respectively, both evaluated in the ${{K} ^+} {{\pi } ^-}$ rest frame. The *X*(*z*) terms are the Blatt–Weisskopf barrier factors [45] reported in Appendix A. The barrier radius, $r_{\text {BW}}$, is taken to be $\,\mathrm {[} 4]{GeV^{-1}}$ (corresponding to $\sim 0.8 \,\mathrm {fm}$) for all resonances. The $Z(\vec {p},\vec {q})$ term describes the angular probability distribution in the Zemach tensor formalism [46, 47], given by the equations reported in Appendix B. The function $T[m({{K} ^+} {{\pi } ^-})]$ of Eq. (6) is the mass lineshape. Most of the resonant contributions are described by the relativistic Breit–Wigner (RBW) function

7 $$\begin{aligned} T(m) = \frac{1}{m_0^2 - m^2-im_0\Gamma (m)}, \end{aligned}$$

where the mass-dependent width is given by

8 $$\begin{aligned} \Gamma (m) = \Gamma _0 \left( \frac{|\vec {q}|}{q_0} \right) ^{(2L+1)} \left( \frac{m_0}{m} \right) X^2(|\vec {q}| r_{\text {BW}}) \end{aligned}$$

and $q_0$ is the value of $|\vec {q}|$ for $m=m_0$, $m_0$ being the pole mass of the resonance.

The amplitude parametrisations using RBW functions lead to unitarity violation within the isobar model if there are overlapping resonances or if there is a significant interference with a NR component, both in the same partial wave [48]. This is the case for the ${{K} ^+} {{\pi } ^-}$ S-wave at low ${{K} ^+} {{\pi } ^-}$ mass, where the $K^*_0(1430)^0$ resonance interferes strongly with a slowly varying NR S-wave component. Therefore, the ${{K} ^+} {{\pi } ^-}$ S-wave at low mass is modelled using a modified LASS lineshape [49], given by

9 $$\begin{aligned} T(m) = \frac{m}{|\vec {q}|\cot \delta _B - i |\vec {q}|} + e^{2i\delta _B}\frac{m_0\Gamma _0\frac{m_0}{q_0}}{m_0^2 - m^2 -im_0\Gamma _0\frac{|\vec {q}|}{m}\frac{m_0}{q_0}}, \end{aligned}$$

with

10 $$\begin{aligned} \cot \delta _B = \frac{1}{a |\vec {q}|} + \frac{1}{2}r |\vec {q}|, \end{aligned}$$

and where $m_0$ and $\Gamma _0$ are the pole mass and width of the $K^*_0(1430)^0$ state, and *a* and *r* are the scattering length and the effective range, respectively. The parameters *a* and *r* depend on the production mechanism and hence on the decay under study. The slowly varying part (the first term in Eq. (9)) is not well modelled at high masses and it is set to zero for $m({{K} ^+} {{\pi } ^-})$ values above $\,\mathrm {[} 1.7]{GeV}$.

The probability density function for signal events across the DP, neglecting reconstruction effects, can be written as

11 $$\begin{aligned}&\mathcal {P}_{\text {sig}}[m^2({{K} ^+} {{\pi } ^-}),m^2({\eta _{c}} {{\pi } ^-})] \nonumber \\&\quad = \frac{|\mathcal {A}|^2}{\int _{\text {DP}} |\mathcal {A}|^2\text {d}m^2({{K} ^+} {{\pi } ^-})~ \text {d}m^2({\eta _{c}} {{\pi } ^-})}, \end{aligned}$$

where the dependence of $\mathcal {A}$ on the DP position has been suppressed for brevity. The natural width of the  meson is set to zero when computing the DP normalisation shown in the denominator of Eq. (11). The effect of this simplification is determined when assessing the systematic uncertainties as described in Sect. 7.

The complex coefficients, given by $c_j$ in Eq. (4), depend on the choice of normalisation, phase convention and amplitude formalism. Fit fractions and interference fit fractions are convention-independent quantities that can be directly compared between different analyses. The fit fraction is defined as the integral of the amplitude for a single component squared divided by that of the coherent matrix element squared for the complete DP,

12 $$\begin{aligned} \text {FF}_i = \frac{\int _{\text {DP}} |c_i\mathcal {F}_i[m^2({{K} ^+} {{\pi } ^-}),m^2({\eta _{c}} {{\pi } ^-})]|^2 \text {d} m^2({{K} ^+} {{\pi } ^-})~\text {d}m^2({\eta _{c}} {{\pi } ^-})}{\int _{\text {DP}} |\mathcal {A}[m^2({{K} ^+} {{\pi } ^-}),m^2({\eta _{c}} {{\pi } ^-})]|^2 \text {d}m^2({{K} ^+} {{\pi } ^-})~\text {d}m^2({\eta _{c}} {{\pi } ^-})}. \end{aligned}$$

In general, the fit fractions do not sum to unity due to the possible presence of net constructive or destructive interference over the whole DP area. This effect can be described by interference fit fractions defined for $i<j$ by

13 $$\begin{aligned} \text {FF}_{ij} = \frac{\int _{\text {DP}} 2 \mathcal {R}e \left[ c_ic_j^*\mathcal {F}_i \mathcal {F}_j^* \right] \text {d}m^2({{K} ^+} {{\pi } ^-})~\text {d}m^2({\eta _{c}} {{\pi } ^-})}{\int _{\text {DP}} |\mathcal {A}|^2\text {d}m^2({{K} ^+} {{\pi } ^-})~\text {d}m^2({\eta _{c}} {{\pi } ^-})}, \end{aligned}$$

where the dependence of $\mathcal {F}_i^{(*)}$ and $\mathcal {A}$ on the DP position is omitted.

## Dalitz plot fit

The Laura $^{++}$ package [50] is used to perform the unbinned DP fit, with the Run 1 and 2 subsamples fitted simultaneously using the JFIT framework [51]. The free parameters in the amplitude fit are in common between the two subsamples, while the signal and background yields and the maps describing the efficiency variations across the phase space, are different. Within the DP fit, the signal corresponds to  decays, while the background comprises both combinatorial background and NR  contributions. The likelihood function is given by

14 $$\begin{aligned} \mathcal {L} = \prod _i^{N_c} \left[ \sum _k N_k \mathcal {P}_k [m_i^{2}(K^+\pi ^-),m_i^{2}(\eta _c \pi ^-)] \right] , \end{aligned}$$

where the index *i* runs over the $N_c$ candidates, *k* runs over the signal and background components, and $N_k$ is the yield of each component. The procedure to determine the signal and background yields is described in Sect. 6.1. The probability density function for the signal, $\mathcal {P}_{\text {sig}}$, is given by Eq. (11) where the $|\mathcal {A}[m^2({{K} ^+} {{\pi } ^-}),m^2({\eta _{c}} {{\pi } ^-})]|^2$ term is multiplied by the efficiency function described in Sect. 6.3. In order to avoid problems related to the imperfect parametrisation of the efficiencies at the DP borders, a veto of $\pm \,\mathrm {[} 70]{MeV}$ is applied around the DP, *i.e.* to the phase space boundaries of the $m({{K} ^+} {{\pi } ^-})$, $m({\eta _{c}} {{\pi } ^-})$ and $m({\eta _{c}} {{K} ^+})$ distributions. This veto is used when determining the signal and background yields, and the probability density functions for the background, obtained as described in Sect. 6.2. The ${{K} ^+} {{\pi } ^-}$ mass resolution is $\approx \,\mathrm {[} 5]{MeV}$, which is much smaller than the $K^*(892)^0$ meson width $\Gamma _{K^*(892)^0} \approx \,\mathrm {[} 50]{MeV}$, the narrowest contribution to the DP; therefore, the resolution has negligible effects and is not considered further. The amplitude fits are repeated many times with randomised initial values to ensure the absolute minimum is found.

### Signal and background yields

There is a non-negligible fraction of NR  decays in the region of the  meson. In order to separate the contributions of  and NR  decays, a two-dimensional (2D) UML fit to the  and $m({p} {\overline{{p}}})$ distributions is performed in the domain  and $2908< m({p} {\overline{{p}}})< 3058\,\mathrm {Me}\mathrm {V}$. These ranges are chosen to avoid the misidentified decays reported in Sect. 4.1, and they also define the DP fit domain. The Run 1 and 2 2D mass fits are performed separately. The  distributions of  signal and NR  decays are described by Hypatia functions. The  distribution of the combinatorial background is parametrised using an exponential function. The $m({p} {\overline{{p}}})$ distribution of  signal decays is described by the same model described in Sect. 4.1. A possible component where genuine  mesons are combined with random kaons and pions from the PV is investigated but found to be negligible. The ${B} ^0$ meson mass, the  resolution, the value of $m_{\eta _c}$, the slopes of the exponential functions, and the yields, are free to vary in the 2D mass fits. The $m({p} {\overline{{p}}})$ resolution and the  meson natural width are Gaussian constrained to the value obtained in the fit to the weighted $m({p} {\overline{{p}}})$ distribution of Sect. 4.1, and to the known value [8], respectively.

**Table 2:** Yields of the components in the 2D mass fit to the joint [, $m({p} {\overline{{p}}})$] distribution for the Run 1 and 2 subsamples

Component	Run 1	Run 2
	$805 \pm 48$	$1065 \pm 56$
(NR)	$234 \pm 48$	$273 \pm 56$
Combinatorial background	$409 \pm 36$	$498 \pm 41$

**Fig. 4:**
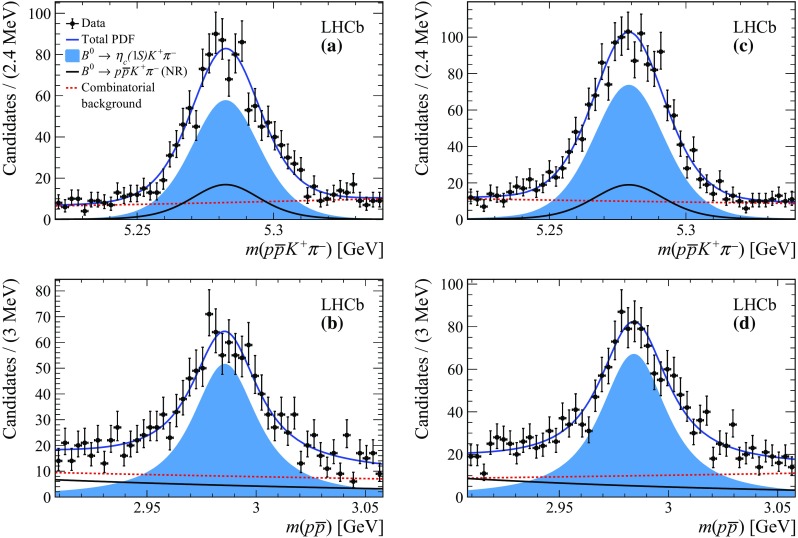
Results of the 2D mass fit to the joint [, $m({p} {\overline{{p}}})$] distribution for the **a** Run 1  projection, **b** Run 1 $m({p} {\overline{{p}}})$ projection, **c** Run 2  projection, and **d** Run 2 $m({p} {\overline{{p}}})$ projection. The legend is shown in the top left plot

The yields of all fit components are reported in Table 2. Figure 4 shows the result of the 2D mass fits for the Run 1 and 2 subsamples that yield a total of approximately 2000  decays. The total yield of the  component is lower than that reported in Sect. 4.1 since the fit ranges are reduced. The goodness of fit is validated using pseudoexperiments to determine the 2D pull, *i.e.* the difference between the fit model and data divided by the uncertainty.

**Fig. 5:**
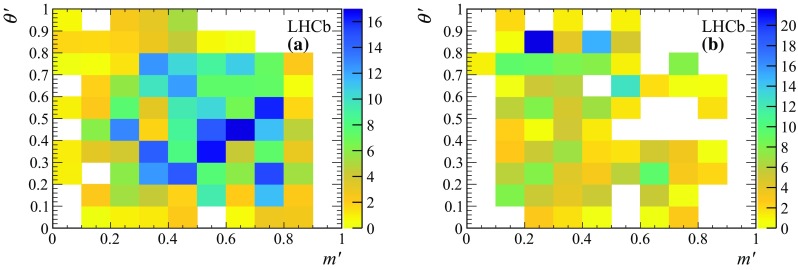
SDP distributions used in the DP fit to the Run 2 subsample for **a** combinatorial background and **b** NR  background

**Fig. 6:**
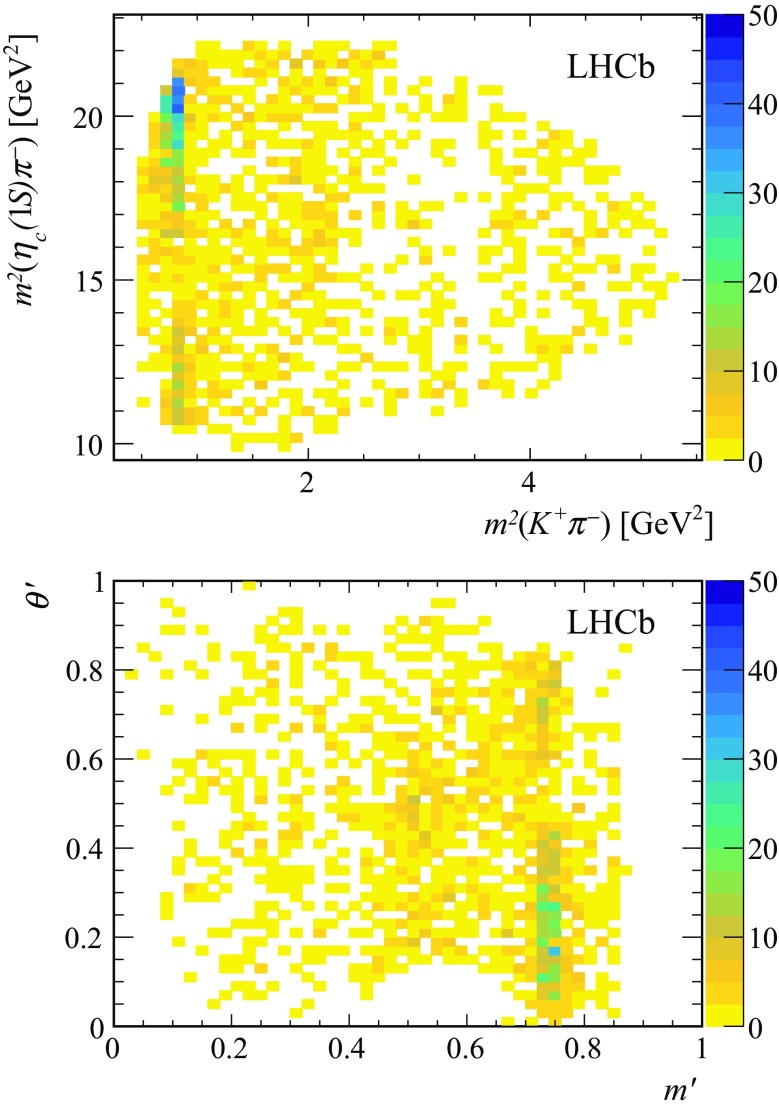
Background-subtracted (top) DP and (bottom) SDP distributions corresponding to the total data sample used in the analysis. The structure corresponding to the $K^*(892)^0$ resonance is evident. The veto of  decays in the ${\overline{D}{}} {}^0$ region is visible in the DP

**Fig. 7:**
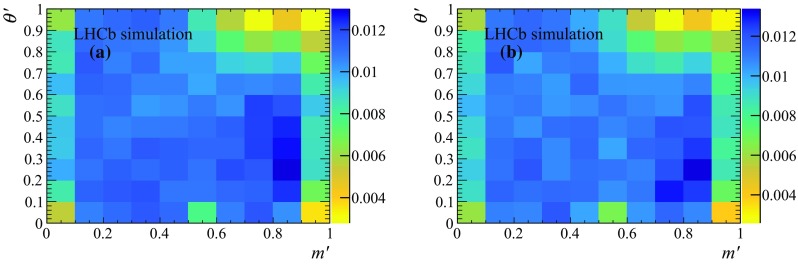
signal efficiency across the SDP for the **a** Run 1 and **b** Run 2 samples

### Parametrisation of the backgrounds

The probability density functions for the combinatorial and NR background categories are obtained from the DP distribution of each background source, represented with a uniformly binned 2D histogram. In order to avoid artefacts related to the curved boundaries of the DP, the histograms are built in terms of the Square Dalitz plot (SDP) parametrised by the variables $m'$ and $\theta '$ which are defined in the range 0 to 1 and are given by

15 $$\begin{aligned} m'&\equiv \frac{1}{\pi } \arccos \left( 2 \frac{m({{K} ^+} {{\pi } ^-})- m^{\min }_{{{K} ^+} {{\pi } ^-}}}{m^{\max }_{{{K} ^+} {{\pi } ^-}} - m^{\min }_{{{K} ^+} {{\pi } ^-}}} - 1\right) , \end{aligned}$$

16 $$\begin{aligned} \theta '&\equiv \frac{1}{\pi } \theta ({{K} ^+} {{\pi } ^-}), \end{aligned}$$

where $\displaystyle m^{\max }_{{{K} ^+} {{\pi } ^-}} = m_{B^0} - m_{\eta _c}$, $\displaystyle m^{\min }_{{{K} ^+} {{\pi } ^-}} = m_{K^+} + m_{\pi ^-}$ are the kinematic boundaries of $m({{K} ^+} {{\pi } ^-})$ allowed in the  decay, and $\displaystyle \theta ({{K} ^+} {{\pi } ^-})$ is the helicity angle of the ${{K} ^+} {{\pi } ^-}$ system (the angle between the $K^+$ and the  mesons in the ${{K} ^+} {{\pi } ^-}$ rest frame).

The combinatorial and NR background histograms are filled using the weights obtained by applying the *sPlot* technique to the joint [, $m({p} {\overline{{p}}})$] distribution, merging the Run 1 and 2 data samples. Each histogram is scaled for the corresponding yield in the two subsamples. The combinatorial and NR background histograms for the Run 2 subsample are shown in Fig. 5. Statistical fluctuations in the histograms due to the limited size of the samples are smoothed by applying a 2D cubic spline interpolation.

The 2D mass fit described in Sect. 6.1 is repeated to the combined Run 1 and 2 data sample, and the *sPlot* technique is applied to determine the background-subtracted DP and SDP distributions shown in Fig. 6.

### Signal efficiency

Efficiency variation across the SDP is caused by the detector acceptance and by the trigger and offline selection requirements. The efficiency variation is evaluated with simulated samples generated uniformly across the SDP. Corrections are applied for known differences between data and simulation in PID efficiencies. The effect of the vetoes in the phase space is separately accounted for by the Laura $^{++}$ package, setting to zero the signal efficiency within the vetoed regions. Therefore, the vetoes corresponding to the ${\overline{D}{}} {}^0$ meson and the phase-space border are not applied when constructing the numerator of the efficiency histogram. The efficiency is studied separately for the Run 1 and 2 subsamples, and the resulting efficiency maps are shown in Fig. 7. Lower efficiency in regions with a low-momentum track is due to geometrical effects. Statistical fluctuations in the histograms due to the limited size of the simulated samples are smoothed by applying a 2D cubic spline interpolation.

### Amplitude model with only ${{K} ^+} {{\pi } ^-}$ contributions

In the absence of contributions from exotic resonances, only ${{K} ^+} {{\pi } ^-}$ resonances are expected as intermediate states. The established ${{K} ^{*0}} \rightarrow {{K} ^+} {{\pi } ^-}$ mesons reported in Ref. [8] with , i.e. with masses within or slightly above the phase space boundary in  decays, are used as a guide when building the model. Only those amplitudes providing significant improvements in the description of the data are included. This model is referred to as the baseline model and comprises the resonances shown in Table 3.

**Table 3:** Resonances included in the baseline model, where parameters and uncertainties are taken from Ref. [52]. The LASS lineshape also parametrise the ${{K} ^+} {{\pi } ^-}$ S-wave in  NR decays

Resonance	Mass $[\,\mathrm {[} ]{MeV}]$	Width $[\,\mathrm {[} ]{MeV}]$	$J^P$	Model
$K^*(892)^0$	$895.55 \pm 0.20$	$47.3 \pm 0.5$	$1^-$	RBW
$K^*(1410)^0$	$1414 \pm 15$	$232 \pm 21$	$1^-$	RBW
$K^*_0(1430)^0$	$1425 \pm 50$	$270 \pm 80$	$0^+$	LASS
$K^*_2(1430)^0$	$1432.4 \pm 1.3$	$109 \pm 5$	$2^+$	RBW
$K^*(1680)^0$	$1717 \pm 27$	$322 \pm 110$	$1^-$	RBW
$K^*_0(1950)^0$	$1945 \pm 22$	$201 \pm 90$	$0^+$	RBW

**Fig. 8:**
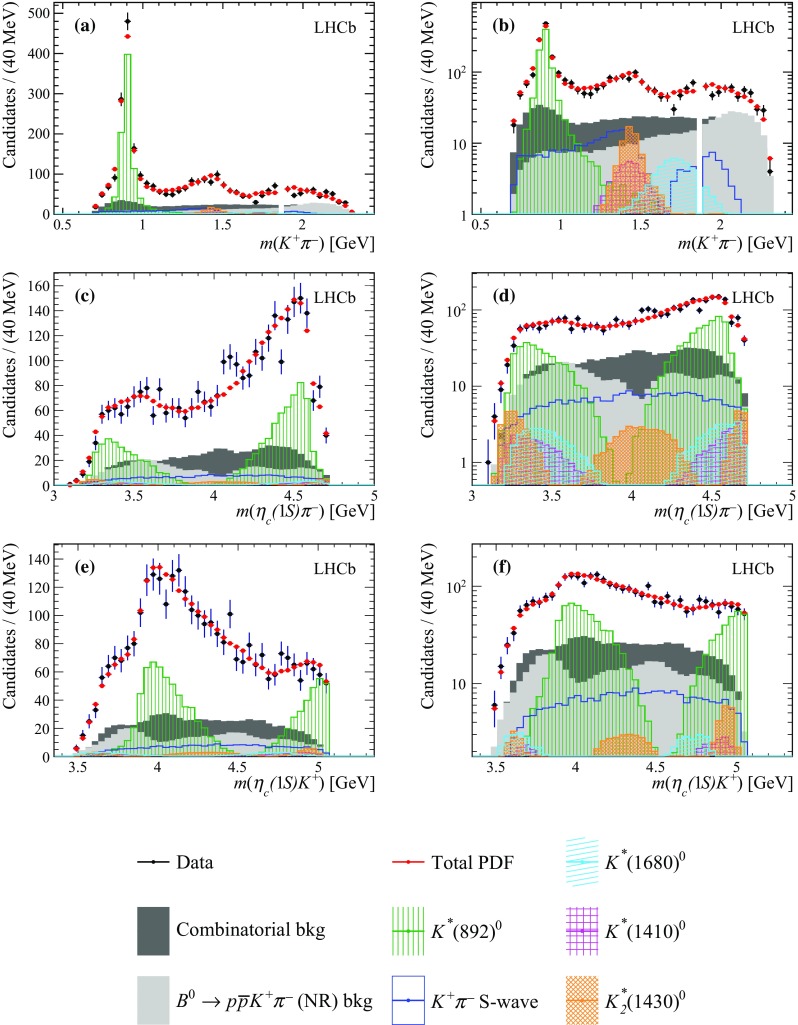
Projections of the data and amplitude fit using the baseline model onto **a** $m({{K} ^+} {{\pi } ^-})$, **c** $m({\eta _{c}} {{\pi } ^-})$ and **e** $m({\eta _{c}} {{K} ^+})$, with the same projections shown in **b**, **d** and **f** with a logarithmic vertical-axis scale. The veto of ${{B} ^0} \!\rightarrow {p} {\overline{{p}}} {{\overline{D}{}} {}^0}$ decays is visible in plot **b**. The ${{K} ^+} {{\pi } ^-}$ S-wave component comprises the LASS and $K^*_0(1950)^0$ meson contributions. The components are described in the legend at the bottom

**Table 4:** Complex coefficients and fit fractions determined from the DP fit using the nominal model. Uncertainties are statistical only

Amplitude	Real part	Imaginary part	Fit fraction (%)
$B^0 \rightarrow \eta _c K^*(892)^0$	1 (fixed)	0 (fixed)	$51.4 \pm 1.9$
$B^0 \rightarrow \eta _c K^*(1410)^0$	$0.17 \pm 0.07$	$0.11 \pm 0.08$	$2.1 \pm 1.1$
$B^0 \rightarrow \eta _c K^+ \pi ^-$ (NR)	$-0.45 \pm 0.08$	$0.01 \pm 0.09$	$10.3 \pm 1.4$
$B^0 \rightarrow \eta _c K^*_0(1430)^0$	$-0.62 \pm 0.09$	$-0.33 \pm 0.25$	$25.3 \pm 3.5$
$B^0 \rightarrow \eta _c K^*_2(1430)^0$	$0.16 \pm 0.06$	$-0.23 \pm 0.05$	$4.1 \pm 1.5$
$B^0 \rightarrow \eta _c K^*(1680)^0$	$-0.11 \pm 0.08$	$-0.18 \pm 0.06$	$2.2 \pm 2.0$
$B^0 \rightarrow \eta _c K^*_0(1950)^0$	$0.27 \pm 0.04$	$0.04 \pm 0.14$	$3.8 \pm 1.8$
$B^0 \rightarrow Z_c(4100)^{-} K^+$	$-0.25 \pm 0.04$	$-0.01 \pm 0.08$	$3.3 \pm 1.1$

The S-wave at low ${{K} ^+} {{\pi } ^-}$ mass is modelled with the LASS probability density function. The real and imaginary parts of the complex coefficients $c_j$ introduced in Eq. (4) are free parameters of the fit, except for the $K^*(892)^0$ component, which is taken as the reference amplitude. Other free parameters in the fit are the scattering length (*a*) and the effective range (*r*) parameters of the LASS function, defined in Eq. (9). The mass and width of the $K^*_0(1430)^0$ meson are Gaussian constrained to the known values [8].

While it is possible to describe the $m({{K} ^+} {{\pi } ^-})$ and $m({\eta _{c}} {{K} ^+})$ distributions well with ${{K} ^+} {{\pi } ^-}$ contributions alone, the fit projection onto the $m({\eta _{c}} {{\pi } ^-})$ distribution does not provide a good description of data, as shown in Fig. 8. In particular, a discrepancy around $m({\eta _{c}} {{\pi } ^-})\approx \,\mathrm {[} 4.1]{GeV}$ is evident.

A $\chi ^2$ variable is computed as a quantitative determination of the fit quality, using an adaptive 2D binning schema to obtain 144 equally populated bins. The baseline model yields a $\chi ^2/\text {ndof}=195/129=1.5$ value, where ndof is the number of degrees of freedom. Including additional ${{K} ^+} {{\pi } ^-}$ resonant states does not lead to significant improvements in the description of the data. These include established states such as the $K^*_3(1780)^0$ and $K^*_4(2045)^0$ mesons, the high mass $K^*_5(2380)^0$ resonance which falls outside the phase space limits, and the $K^*_2(1980)^0$ state which has not been seen in the ${{K} ^+} {{\pi } ^-}$ final state thus far. The unestablished P-, D- and F-wave ${{K} ^+} {{\pi } ^-}$ states predicted by the Godfrey–Isgur model [53] to decay into the ${{K} ^+} {{\pi } ^-}$ final state were also tested.

### Amplitude model with ${{K} ^+} {{\pi } ^-}$ and  contributions

A better description of the data is obtained by adding an exotic  component to the ${{K} ^+} {{\pi } ^-}$ contributions of Table 3. The resulting signal model consists of eight amplitudes: seven resonances and one NR term. The ${{K} ^+} {{\pi } ^-}$ amplitudes are modelled in the same way as in the baseline model. Alternative models for the ${{K} ^+} {{\pi } ^-}$ S-wave are used to assign systematic uncertainties as discussed in Sect. 7. In addition to the free parameters used in the baseline model, the isobar coefficients, mass and width of the $Z^-_c$ resonance are left floating.

A likelihood-ratio test is used to discriminate between any pair of amplitude models based on the log-likelihood difference $\Delta ( -2 \ln \mathcal {L})$ [54]. Three quantum number hypotheses are probed for the $Z_c^-$ resonance, repeating the amplitude fit for the $J^P=0^+~,1^-~\text {and}~2^+$ assignments. The variations of the $\Delta ( -2 \ln \mathcal {L})$ value with respect to the baseline model are $\Delta ( -2 \ln \mathcal {L})=22.8,~41.4$, and 7.0, respectively. The model providing the best description of the data, referred to below as the nominal fit model, is obtained with the addition of a $Z^-_c$ candidate with $J^P=1^-$. The $J^P=2^+$ assignment is not considered further given the small variation in $\ln \mathcal {L}$ with respect to the additional four free parameters.

The LASS parameters obtained in the nominal fit model are $m_{K^{*}_0(1430)^0} = \,\mathrm {[} 1427 \pm 21]{MeV}$, $\Gamma _{K^{*}_0(1430)^0} = \,\mathrm {[} 256 \pm 33]{MeV}$, $a = \,\mathrm {[} 3.1 \pm 1.0]{GeV^{-1}}$ and $r = \,\mathrm {[} 7.0 \pm 2.4]{GeV^{-1}}$. The parameters of the $Z_c^-$ candidate obtained in the nominal fit model are $m_{Z_c^-} =\,\mathrm {[} 4096 \pm 20]{MeV}$ and $\Gamma _{Z_c^-} = \,\mathrm {[} 152 \pm 58]{MeV}$. The values of the complex coefficients and fit fractions returned by the nominal fit model are shown in Table 4. The statistical uncertainties on all parameters of interest are calculated using large samples of simulated pseudoexperiments generated from the fit results in order to take into account the correlations between parameters and to guarantee the correct coverage of the uncertainties.

**Fig. 9:**
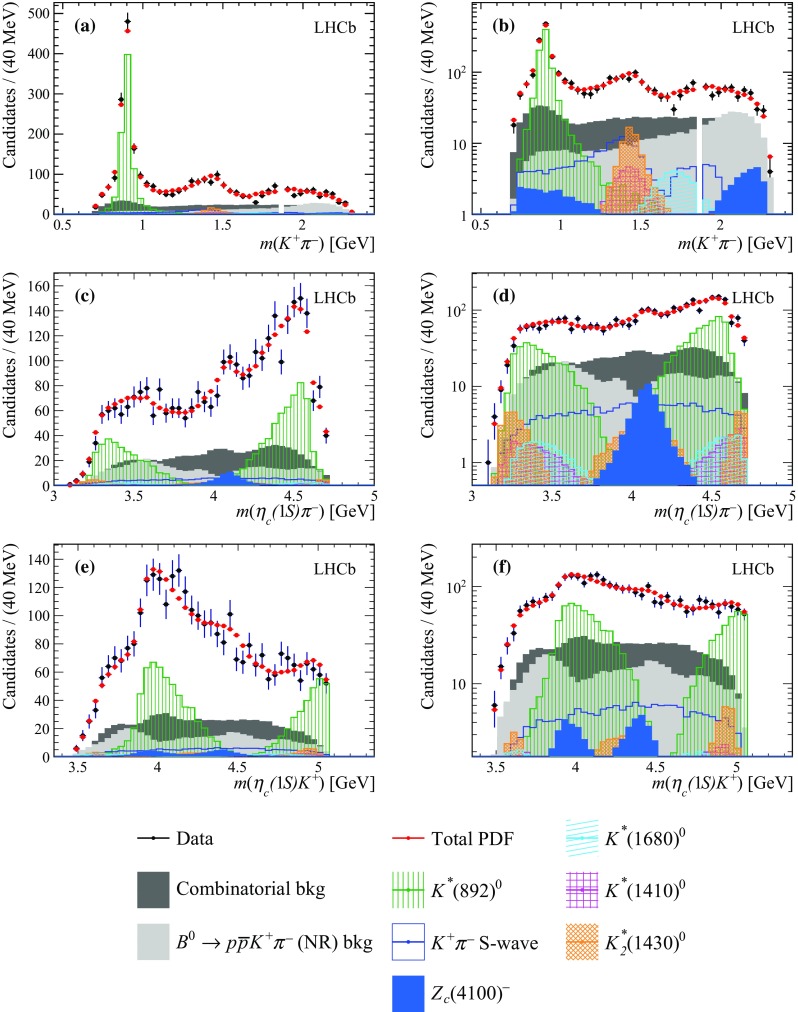
Projections of the data and amplitude fit using the nominal model onto **a** $m({{K} ^+} {{\pi } ^-})$, **c** $m({\eta _{c}} {{\pi } ^-})$ and **e** $m({\eta _{c}} {{K} ^+})$, with the same projections shown in **b**, **d** and **f** with a logarithmic vertical-axis scale. The veto of ${{B} ^0} \!\rightarrow {p} {\overline{{p}}} {{\overline{D}{}} {}^0}$ decays is visible in plot **b**. The ${{K} ^+} {{\pi } ^-}$ S-wave component comprises the LASS and $K^*_0(1950)^0$ meson contributions. The components are described in the legend at the bottom

Figure 9 shows the projections of the nominal fit model and the data onto $m({{K} ^+} {{\pi } ^-})$, $m({\eta _{c}} {{\pi } ^-})$ and $m({\eta _{c}} {{K} ^+})$ invariant masses. A good agreement between the nominal fit model and the data is obtained. The value of the $\chi ^2/\text {ndof}$ is $164/125=1.3$ for the nominal fit model. The fit quality is further discussed in Appendix C, where a comparison is reported of the unnormalised Legendre moments between data, the baseline and nominal models. The 2D pull distributions for the baseline and nominal models are reported as well.

The significance of the $Z_c^-$ candidate, referred to as the $Z_c(4100)^{-}$ state in the following, is evaluated from the change in the likelihood of the fits with and without the $Z_c(4100)^{-}$ component, assuming that this quantity, $\Delta ( -2 \ln \mathcal {L})$, follows a $\chi ^2$ distribution with a number of degrees of freedom equal to twice the number of free parameters in its parametrisation [17, 55–57]. This assumption takes into account the look-elsewhere effect due to the floating mass and width of the $Z_c(4100)^-$. The validity of this assumption is verified using pseudoexperiments to predict the distribution of $\Delta ( -2 \ln \mathcal {L})$ under the no-$Z_c(4100)^{-}$ hypothesis, which is found to be well described by a $\chi ^2$ probability density function with ndof $=$ 8. The statistical significance of the $Z_c(4100)^{-}$ is $4.8\sigma$ in the nominal fit model. The quoted significance does not include the contribution from systematic uncertainties.

To discriminate between various $J^P$ assignments, fits are performed under alternative $J^P$ hypotheses. A lower limit on the significance of rejection of the $J^P=0^+$ hypothesis is determined from the change in the log-likelihood from the preferred hypothesis, assuming a $\chi ^2$ distribution with one degree of freedom. The validity of this assumption is verified using pseudoexperiments to predict the distribution of $\Delta ( -2 \ln \mathcal {L})$ under the disfavoured $J^P=0^+$ hypothesis. The statistical rejection of the $J^P=0^+$ hypothesis with respect to the $J^P=1^-$ hypothesis is $4.3\sigma$.

Systematic effects must be taken into account to report the significance of the $Z_c(4100)^-$ contribution and the discrimination of its quantum numbers. The fit variations producing the largest changes in the values of the mass, width or isobar coefficients of the exotic candidate are used to probe the sensitivity of the significance of the $Z_c(4100)^{-}$ state to systematic effects, and to determine its quantum numbers, as described in Sect. 7.

## Systematic uncertainties

Systematic uncertainties can be divided into two categories: experimental and model uncertainties. Among the experimental uncertainties, the largest changes in the values of the parameters of the $Z_c(4100)^{-}$ candidate are due to the signal and background yields used in the amplitude fit, the SDP distributions of the background components, and the phase-space border veto applied on the parametrisation of the efficiencies. Among the model uncertainties, the largest effects are due to the treatment of the natural width of the  meson within the DP fit and to the ${{K} ^+} {{\pi } ^-}$ S-wave parametrisation. The DP fits using the baseline and nominal varied models are used to recompute the significance.

The signal and background yields used in the amplitude fit are fixed to the values obtained from the 2D mass fit. The statistical uncertainties on the yields are introduced into the amplitude fit by Gaussian constraining the yields within their statistical uncertainties and by repeating the fit.

The systematic uncertainties associated to the parametrisation of the background distributions are evaluated by varying the value in each bin within the statistical uncertainty prior to the spline interpolation. About 300 new background histograms are produced for both the combinatorial and NR background components. The resulting $\Delta ( -2 \ln \mathcal {L})$ distribution follows a Gaussian distribution. The most pessimistic background parametrisation, corresponding to a $\Delta ( -2 \ln \mathcal {L})$ value that is below $3\sigma$ of the Gaussian distribution, is considered when quoting the effect of this source on the significance of the $Z_c(4100)^-$ state.

The phase-space border veto applied on the parametrisation of the efficiencies is removed to check the veto does not significantly affect the result.

The natural width of the  meson is set to zero when computing the DP normalisations, calculated using the  meson mass values resulting from the 2D UML fits described in Sect. 6.1. In order to associate a systematic uncertainty to the sizeable  natural width, the amplitude fits are repeated computing the DP normalisations by using the  and  values, where  and  are the mass and natural width of the  meson, respectively, obtained from the 2D UML fits.

The LASS model used to parametrise the low mass ${{K} ^+} {{\pi } ^-}$ S-wave in the nominal fit is replaced with $K^*_0(1430)^0$ and $K^*_0(700)^0$ resonances parametrised with RBW functions, and a NR S-wave ${{K} ^+} {{\pi } ^-}$ component parametrised with a uniform amplitude within the DP.

The effect of the separate systematic sources to the significance of the $Z_c(4100)^-$ are reported in Table 5. When including the most important systematic effect, corresponding to the pessimistic background parametrisation, the lowest significance for the $Z_c(4100)^-$ candidate is given by $3.4\sigma$. In order to evaluate the effect of possible correlated or anti-correlated sources of systematic uncertainty, the fits are repeated using the pessimistic background parametrisation together with the alternative ${{K} ^+} {{\pi } ^-}$ S-wave model, and with mass values of the  meson varied within the corresponding statistical uncertainty resulting from the 2D UML fit. The lower limit on the significance of the $Z_c(4100)^-$ state is found to be $3.2\sigma$.

**Table 5:** Significance of the $Z_c(4100)^-$ contribution for the systematic effects producing the largest variations in the parameters of the $Z_c(4100)^-$ candidate. The values obtained in the nominal amplitude fit are shown in the first row

Source	$\Delta ( -2 \ln \mathcal {L})$	Significance
Nominal fit	41.4	$4.8\sigma$
Fixed yields	45.8	$5.2\sigma$
Phase-space border veto	44.6	$5.1\sigma$
width	36.6	$4.3\sigma$
${{K} ^+} {{\pi } ^-}$ S-wave	31.8	$3.9\sigma$
Background	27.4	$3.4\sigma$

**Table 6:** Rejection level of the $J^P=0^+$ hypothesis with respect to the $J^P=1^-$ hypothesis for the systematic variations producing the largest variations in the parameters of the $Z_c(4100)^-$ candidate. The values obtained in the nominal amplitude fit are shown in the first row

Source	$\Delta ( -2 \ln \mathcal {L})$	Significance
Default	18.6	$4.3\sigma$
Fixed yields	23.8	$4.9\sigma$
Phase-space border veto	24.4	$4.9\sigma$
width	4.2	$2.0\sigma$
Background	3.4	$1.8\sigma$
${{K} ^+} {{\pi } ^-}$ S-wave	1.4	$1.2\sigma$

The discrimination between the $J^P=0^+$ and $J^P=1^-$ assignments is not significant when systematic uncertainties are taken into account, as reported in Table 6. When the S-wave model is varied, the two spin-parity hypotheses only differ by $1.2\sigma$.

Additional sources of systematic uncertainties are considered when evaluating the uncertainty on the mass and width of the $Z_c(4100)^{-}$ resonance, and on the fit fractions obtained with the nominal model. These additional sources are: the efficiency variation across the SDP and a possible bias due to the fitting procedure, contributing to the experimental systematic uncertainties category; and the fixed parameters of the resonances in the amplitude model and the addition or removal of marginal amplitudes, contributing to the model systematic uncertainties category. For each source, the systematic uncertainty assigned to each quantity is taken as the difference between the value returned by the modified amplitude fit and nominal model fit result. The uncertainties due to all these sources are obtained by combining positive and negative deviations in quadrature separately.

The bin contents of the histograms describing the efficiency variation across the SDP are varied within their uncertainties prior to the spline interpolation, as is done for the systematic uncertainty associated to the background parametrisations. A possible source of systematic effects in the efficiency histograms is due to neighbouring bins varying in a correlated way. In order to evaluate this systematic uncertainty, 10 bins of the efficiency histograms are varied within their statistical uncertainty, and the neighbouring bins are varied by linear interpolation. The binning scheme of the control sample used to evaluate the PID performance is varied.

Pseudoexperiments are generated from the fit results using the nominal model in order to assign a systematic uncertainty due to possible amplitude fit bias.

Systematic uncertainties due to fixed parameters in the fit model are determined by repeating the fit and varying these parameters. The fixed masses and widths of the ${{K} ^+} {{\pi } ^-}$ contributions are varied 100 times assigning a random number within the range defined by the corresponding uncertainties reported in Table 3. The Blatt–Weisskopf barrier radii, $r_{\text {BW}}$, are varied independently for ${{K} ^+} {{\pi } ^-}$ and  resonances between 3 and $\,\mathrm {[} 5]{GeV^{-1}}$.

Systematic uncertainties are assigned from the changes in the results when the amplitudes due to the established $K^*_3(1780)^0$ and $K^*_4(2045)^0$ resonances, not contributing significantly in the baseline and nominal models, are included.

The total systematic uncertainties for the fit fractions are given together with the results in Sect. 8. The dominant experimental systematic uncertainty is due to either the phase-space border veto, related to the efficiency parametrisation, or the background distributions across the SDP, while the model uncertainties are dominated by the description of the ${{K} ^+} {{\pi } ^-}$ S-wave.

The stability of the fit results is confirmed by several cross-checks. The addition of further high-mass $K^{*0}$ states to the nominal model does not improve the quality of the fit. An additional amplitude decaying to  is not significant, nor is an additional exotic amplitude decaying to . The  meson resonant phase motion due to the sizeable natural width could affect the overall amplitude of Eq. (4), introducing interference effects with the NR $p\bar{p}$ contribution. In order to investigate this effect, the data sample is divided in two parts, containing candidates with masses below and above the  meson peak, respectively. The results are compatible with those reported in Sect. 6 using the full data sample, supporting the argument that the effects due to the variation of the  phase are negligible.

**Table 7:** Fit fractions and their uncertainties. The quoted uncertainties are statistical and systematic, respectively

Amplitude	Fit fraction (%)
$B^0 \rightarrow \eta _c K^*(892)^0$	$51.4 \pm 1.9~^{+1.7}_{-4.8}$
$B^0 \rightarrow \eta _c K^*(1410)^0$	$2.1 \pm 1.1~^{+1.1}_{-1.1}$
$B^0 \rightarrow \eta _c K^+ \pi ^-$ (NR)	$10.3 \pm 1.4~^{+1.0}_{-1.2}$
$B^0 \rightarrow \eta _c K^*_0(1430)^0$	$25.3 \pm 3.5~^{+3.5}_{-2.8}$
$B^0 \rightarrow \eta _c K^*_2(1430)^0$	$4.1 \pm 1.5~^{+1.0}_{-1.6}$
$B^0 \rightarrow \eta _c K^*(1680)^0$	$2.2 \pm 2.0~^{+1.5}_{-1.7}$
$B^0 \rightarrow \eta _c K^*_0(1950)^0$	$3.8 \pm 1.8~^{+1.4}_{-2.5}$
$B^0 \rightarrow Z_c(4100)^- K^+$	$3.3 \pm 1.1~^{+1.2}_{-1.1}$

**Table 8:** Branching fraction results. The four quoted uncertainties are statistical,  branching fraction systematic (not including the contribution from the uncertainty associated to the efficiency ratio, to avoid double counting the systematic uncertainty associated to the evaluation of the efficiencies), fit fraction systematic and external branching fractions uncertainties, respectively

Decay mode	Branching fraction ($10^{-5}$)
$B^0 \rightarrow \eta _c K^*(892)^0 ( \rightarrow K^+\pi ^-)$	$29.5 \pm 1.6 \pm 0.6 \,\,^{+1.0}_{-2.8} \pm 3.4$
$B^0 \rightarrow \eta _c K^*(1410)^0 ( \rightarrow K^+\pi ^-)$	$1.20 \pm 0.63 \pm 0.02 \pm 0.63 \pm 0.14$
$B^0 \rightarrow \eta _c K^+ \pi ^-$ (NR)	$5.90 \pm 0.84 \pm 0.11\,\,^{+0.57}_{-0.69} \pm 0.68$
$B^0 \rightarrow \eta _c K^*_0(1430)^0 ( \rightarrow K^+\pi ^-)$	$14.50 \pm 2.10 \pm 0.28\,\,^{+2.01}_{-1.60} \pm 1.67$
$B^0 \rightarrow \eta _c K^*_2(1430)^0 ( \rightarrow K^+\pi ^-)$	$2.35 \pm 0.87 \pm 0.05\,\,^{+0.57}_{-0.92} \pm 0.27$
$B^0 \rightarrow \eta _c K^*(1680)^0 ( \rightarrow K^+\pi ^-)$	$1.26 \pm 1.15 \pm 0.02 ~^{+0.86}_{-0.97} \pm 0.15$
$B^0 \rightarrow \eta _c K^*_0(1950)^0 ( \rightarrow K^+\pi ^-)$	$2.18 \pm 1.04 \pm 0.04\,\,^{+0.80}_{-1.43} \pm 0.25$
$B^0 \rightarrow Z_c(4100)^- K^+$	$1.89 \pm 0.64 \pm 0.04\,\,^{+0.69}_{-0.63} \pm 0.22$

**Table 9:** Symmetric matrix of the fit fractions (%) from the amplitude fit using the nominal model. The quoted uncertainties are statistical and systematic, respectively. The diagonal elements correspond to the values reported in Table 7

	$K^*(892)^0$	$K^*(1410)^0$	LASS NR	$K^*_0(1430)^0$	$K^*_2(1430)^0$	$K^*(1680)^0$	$K^*_0(1950)^0$	$Z_c(4100)^-$
$K^*(892)^0$	$51.4 \pm 1.9~^{+1.7}_{-4.8}$	$1.7 \pm 1.9~^{+2.4}_{-1.4}$	0	0	0	$-2.1 \pm 1.1~^{+1.4}_{-1.5}$	0	$1.4 \pm 1.0~^{+1.2}_{-1.1}$
$K^*(1410)^0$		$2.1 \pm 1.1~^{+1.1}_{-1.1}$	0	0	0	$-2.5 \pm 1.6~^{+1.9}_{-1.7}$	0	$-0.4 \pm 0.4~^{+0.7}_{-0.5}$
LASS NR			$10.3 \pm 1.4~^{+1.0}_{-1.2}$	$-5.8 \pm 1.3~^{+2.2}_{-2.0}$	0	0	$-3.2 \pm 2.8~^{+4.9}_{-1.4}$	$1.11 \pm 0.23~^{+0.54}_{-0.35}$
$K^*_0(1430)^0$				$25.3 \pm 3.5~^{+3.5}_{-2.8}$	0	0	$4.7 \pm 0.7~^{+1.3}_{-1.5}$	$2.8 \pm 0.4~^{+0.6}_{-0.4}$
$K^*_2(1430)^0$					$4.1 \pm 1.5~^{+1.0}_{-1.6}$	0	0	$0.00 \pm 0.31~^{+0.76}_{-0.26}$
$K^*(1680)^0$						$2.2 \pm 2.0~^{+1.5}_{-1.7}$	0	$0.7 \pm 0.5~^{+0.5}_{-0.9}$
$K^*_0(1950)^0$							$3.8 \pm 1.8~^{+1.4}_{-2.5}$	$0.6 \pm 0.5~^{+0.8}_{-1.1}$
$Z_c(4100)^-$								$3.3 \pm 1.1~^{+1.2}_{-1.1}$

## Results and summary

In summary, the first measurement of the  branching fraction is reported and giveswhere the first uncertainty is statistical, the second systematic, and the third is due to limited knowledge of external branching fractions. The first Dalitz plot analysis of the  decay is performed. A good description of data is obtained when including a charged charmonium-like resonance decaying to the  final state with $m_{Z_c^-} = \,\mathrm {[} 4096 \pm 20~ ^{+18}_{-22}]{MeV}$ and $\Gamma _{Z_c^-}=\,\mathrm {[} 152 \pm 58~^{+60}_{-35}]{MeV}$. The fit fractions are reported in Table 7. The fit fractions for resonant and nonresonant contributions are converted into quasi-two-body branching fractions by multiplying by the  branching fraction. The corresponding results are shown in Table 8. The $B^0 \rightarrow \eta _c K^*(892)^0$ branching fraction is compatible with the world-average value [8], taking into account the $K^*(892)^0 \rightarrow K^+\pi ^-$ branching fraction. The values of the interference fit fractions are given in Table 9.

The significance of the $Z_c(4100)^-$ candidate is more than three standard deviations when including systematic uncertainties. This is the first evidence for an exotic state decaying into two pseudoscalars. The favoured spin-parity assignments, $J^P=0^+$ and $J^P=1^-$, cannot be discriminated once systematic uncertainties are taken into account, which prohibits unambiguously assigning the $Z_c(4100)^-$ as one of the states foreseen by the models described in Sect. 1. Furthermore, the mass value of the $Z_c(4100)^-$ charmonium-like state is above the open-charm threshold, in contrast with the predictions of such models. More data will be required to conclusively determine the nature of the $Z_c(4100)^-$ candidate.

**Fig. 10:**
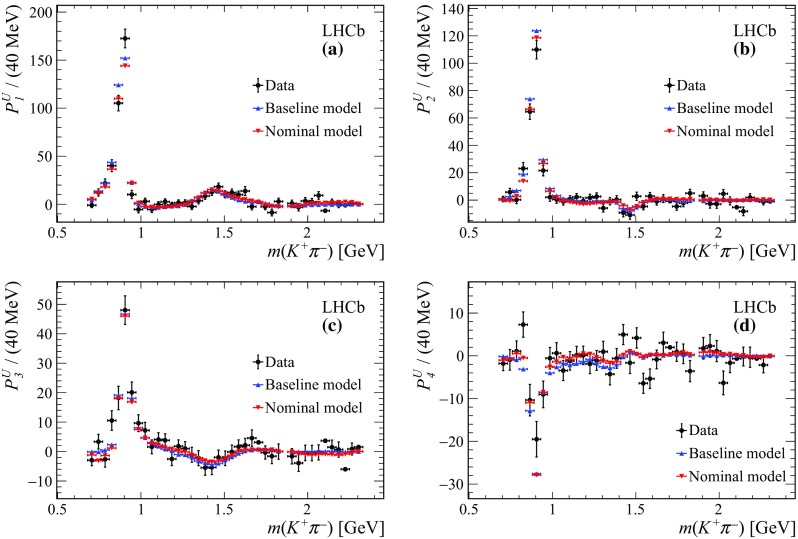
Comparison of the first four ${{K} ^+} {{\pi } ^-}$ Legendre moments determined from background-subtracted data (black points) and from the results of the amplitude fit using the baseline model (red triangles) and nominal model (blue triangles) as a function of $m({{K} ^+} {{\pi } ^-})$

## Figures and Tables

**Fig. 11:**
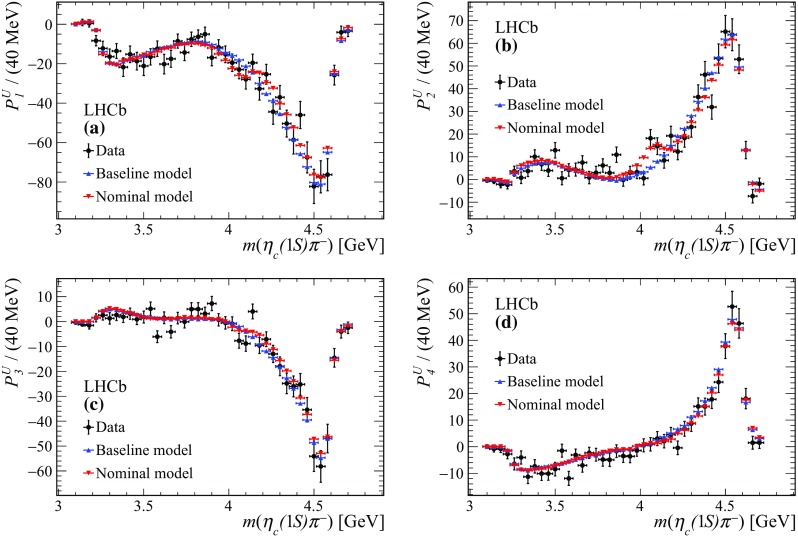
Comparison of the first four  Legendre moments determined from background-subtracted data (black points) and from the results of the amplitude fit using the baseline model (red triangles) and nominal model (blue triangles) as a function of $m({\eta _{c}} {{\pi } ^-})$

**Fig. 12:**
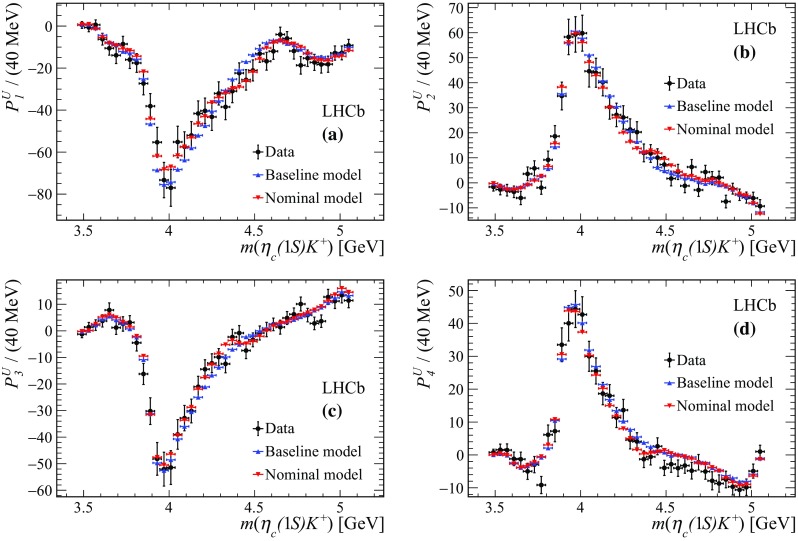
Comparison of the first four  Legendre moments determined from background-subtracted data (black points) and from the results of the amplitude fit using the baseline model (red triangles) and nominal model (blue triangles) as a function of $m({\eta _{c}} {{K} ^+})$

**Fig. 13:**
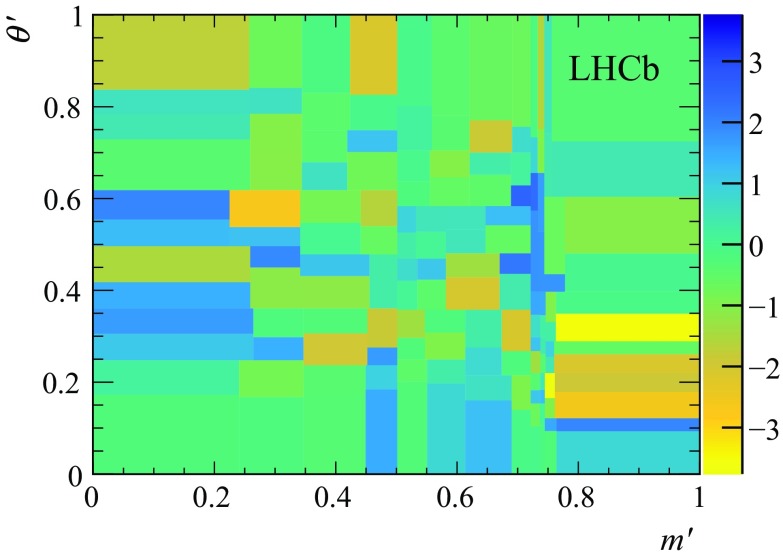
2D pull distribution for to the baseline model

**Fig. 14:**
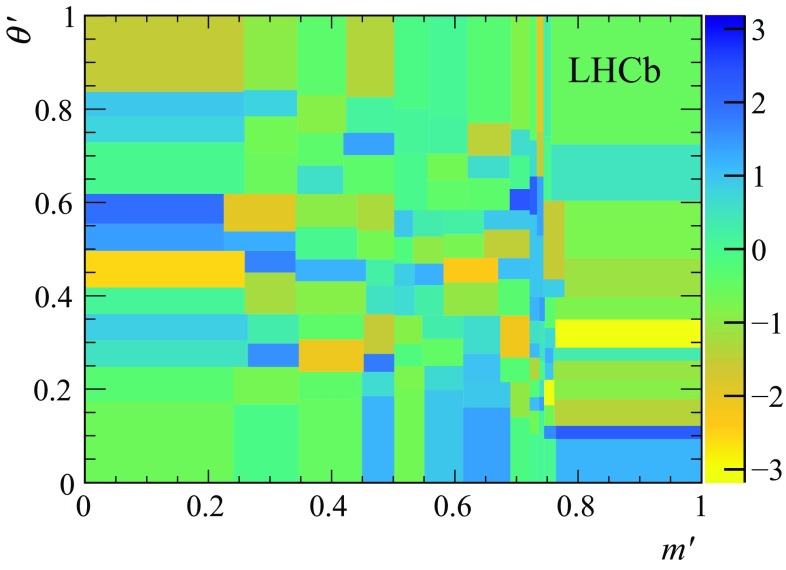
2D pull distribution for to the nominal model

## Appendix: A Blatt–Weisskopf barrier factors

The Blatt–Weisskopf barrier factors [45] *X*(*z*), where $z=|\vec {q}|r_{\text {BW}}$ or $|\vec {p}|r_{\text {BW}}$ with $r_{\text {BW}}$ being the barrier radius, are given by

17 $$\begin{aligned} L&=0:~X(z)=1, \end{aligned}$$

18 $$\begin{aligned} L&=1:~X(z)={\sqrt{\frac{1+z_0^2}{1+z^2}}},\end{aligned}$$

19 $$\begin{aligned} L&=2:~X(z)={\sqrt{\frac{z_0^4+3z_0^2+9}{z^4+3z^2+9}}},\end{aligned}$$

20 $$\begin{aligned} L&=3:~X(z)={\sqrt{\frac{z_0^6+6z_0^4+45z_0^2+225}{z^6+6z^4+45z^2+225}}},\end{aligned}$$

21 $$\begin{aligned} L&=4:~X(z)={\sqrt{\frac{z_0^8+10z_0^6+135z_0^4+1575z_0^2+11025}{z^8+10z^6+135z^4+1575z^2+11025}}}, \end{aligned}$$

where $z_0$ is the value of *z* when the invariant mass is equal to the pole mass of the resonance and *L* is the orbital angular momentum between the resonance children. Since the latter are scalars, *L* is equal to the spin of the resonance. Since the ${B} ^0$ meson and the accompanying particle in the decay are scalars as well, *L* is also equal to the orbital angular momentum between the resonance and the accompanying particle in the decay.

## B: Angular probability distributions

Using the Zemach tensor formalism [46, 47], the angular probability distributions $Z(\vec {p},\vec {q})$ are given by

22 $$\begin{aligned} L&=0:~Z(\vec {p},\vec {q})=1, \end{aligned}$$

23 $$\begin{aligned} L&=1:~Z(\vec {p},\vec {q})=-2\vec {p}\cdot \vec {q},\end{aligned}$$

24 $$\begin{aligned} L&=2:~Z(\vec {p},\vec {q})=\frac{4}{3}\left[ 3(\vec {p}\cdot \vec {q})^2 - (|\vec {p}||\vec {q}|)^2 \right] ,\end{aligned}$$

25 $$\begin{aligned} L&=3:~Z(\vec {p},\vec {q})=-\frac{8}{5}\left[ 5(\vec {p}\cdot \vec {q})^3 - 3(\vec {p}\cdot \vec {q})(|\vec {p}||\vec {q}|)^2 \right] ,\end{aligned}$$

26 $$\begin{aligned} L&=4:~Z(\vec {p},\vec {q})=\frac{16}{35}\left[ 35(\vec {p}\cdot \vec {q})^4 - 30(\vec {p}\cdot \vec {q})^2(|\vec {p}||\vec {q}|)^2 \right. \nonumber \\&\quad \left. + 3(|\vec {p}||\vec {q}|)^4 \right] , \end{aligned}$$

## C: Investigation of the fit quality

Comparisons of the first four Legendre moments determined from background-subtracted data and from the amplitude fit results using the baseline and nominal model are reported in Figs. 10, 11 and 12 for the $m({{K} ^+} {{\pi } ^-})$, $m({\eta _{c}} {{\pi } ^-})$ and $m({\eta _{c}} {{K} ^+})$ projections, respectively.

The 2D pull distributions for the baseline and nominal models are reported in Figs. 13 and 14, respectively.

## Footnotes

1 The *X*(3872) state has been recently renamed $\chi _{c1}(3872)$ in Ref. [8].

2 Natural units with $\hbar = c = 1$ and the simplified notation ${\eta _{c}}$ to refer to the $\eta _c(1S)$ state are used throughout. In addition, the inclusion of charge-conjugate processes is always implied.
